# Prediction of combination therapies based on topological modeling of the immune signaling network in multiple sclerosis

**DOI:** 10.1186/s13073-021-00925-8

**Published:** 2021-07-16

**Authors:** Marti Bernardo-Faura, Melanie Rinas, Jakob Wirbel, Inna Pertsovskaya, Vicky Pliaka, Dimitris E. Messinis, Gemma Vila, Theodore Sakellaropoulos, Wolfgang Faigle, Pernilla Stridh, Janina R. Behrens, Tomas Olsson, Roland Martin, Friedemann Paul, Leonidas G. Alexopoulos, Pablo Villoslada, Julio Saez-Rodriguez

**Affiliations:** 1 European Molecular Biology Laboratory, European Bioinformatics Institute, Cambridge, UK; 2 Centre for Research in Agricultural Genomics (CRAG), CSIC-IRTA-UAB-UB, Campus UAB, Bellaterra, Barcelona, Spain; 3 Joint Research Center for Computational Biomedicine (JRC-COMBINE), Faculty of Medicine, RWTH-Aachen University, Aachen, Germany; 4 Institut d’ Investigacions Biomèdiques August Pi Sunyer (IDIBAPS), Barcelona, Spain; 5 School of Mechanical Engineering, National Technical University of Athens, Zografou, Greece; 6 ProtATonce Ltd., Athens, Greece; 7 University of Zurich, Zurich, Switzerland; 8 Department of Clinical Neuroscience, Karolinska Institutet, Stockholm, Sweden; 9 NeuroCure Clinical Research Center and Department of Neurology, Charité University Medicine Berlin, Berlin, Germany; 10 Institute for Computational Biomedicine, Heidelberg University Hospital and Faculty of Medicine, Heidelberg University, Bioquant, Heidelberg, Germany

**Keywords:** Signaling networks, Pathways, Network modeling, Logic modeling, Kinases, Treatment, Personalized medicine, Combination therapy, Multiple sclerosis, Immunotherapy, Phosphoproteomics, xMAP assay

## Abstract

**Background:**

Multiple sclerosis (MS) is a major health problem, leading to a significant disability and patient suffering. Although chronic activation of the immune system is a hallmark of the disease, its pathogenesis is poorly understood, while current treatments only ameliorate the disease and may produce severe side effects.

**Methods:**

Here, we applied a network-based modeling approach based on phosphoproteomic data to uncover the differential activation in signaling wiring between healthy donors, untreated patients, and those under different treatments. Based in the patient-specific networks, we aimed to create a new approach to identify drug combinations that revert signaling to a healthy-like state. We performed ex vivo multiplexed phosphoproteomic assays upon perturbations with multiple drugs and ligands in primary immune cells from 169 subjects (MS patients, n=129 and matched healthy controls, n=40). Patients were either untreated or treated with fingolimod, natalizumab, interferon-β, glatiramer acetate, or the experimental therapy epigallocatechin gallate (EGCG). We generated for each donor a dynamic logic model by fitting a bespoke literature-derived network of MS-related pathways to the perturbation data. Last, we developed an approach based on network topology to identify deregulated interactions whose activity could be reverted to a “healthy-like” status by combination therapy. The experimental autoimmune encephalomyelitis (EAE) mouse model of MS was used to validate the prediction of combination therapies.

**Results:**

Analysis of the models uncovered features of healthy-, disease-, and drug-specific signaling networks. We predicted several combinations with approved MS drugs that could revert signaling to a healthy-like state. Specifically, TGF-β activated kinase 1 (TAK1) kinase, involved in Transforming growth factor β-1 proprotein (TGF-β), Toll-like receptor, B cell receptor, and response to inflammation pathways, was found to be highly deregulated and co-druggable with all MS drugs studied. One of these predicted combinations, fingolimod with a TAK1 inhibitor, was validated in an animal model of MS.

**Conclusions:**

Our approach based on donor-specific signaling networks enables prediction of targets for combination therapy for MS and other complex diseases.

**Supplementary Information:**

The online version contains supplementary material available at 10.1186/s13073-021-00925-8.

## Background

The signal transduction machinery is frequently affected by perturbations induced by complex diseases. Hence, treatment of diseases such as cancer, cardiovascular, immunological, or brain diseases is nowadays largely attempted by modulating different molecular cascades involved in the disease to stop its progression. As such, kinases involved in signaling processes have evolved as primary targets for many diseases [1]. Further, there has been modest progress with treatments based on single drugs. Combining several drugs targeting different pathways promises more effective modulation of the pathogenic process [2, 3]. However, development of combination therapies is hampered by the often incomplete understanding on how their effect propagates through complex signaling networks, with crosstalk between the pathways influenced by each therapy [4, 5]. As an additional level of complication, disease heterogeneity hinders predicting how a specific combination therapy could be translated into the clinic. Last, the combinatorial nature of such studies in terms of number of targets, drugs, doses, and therapeutic regimens implies a large number of experiments and associated costs, preventing a complete analysis for all alternatives. As a result, the full potential of combination therapies has not been fully developed yet.

Systems biology, and more specifically modeling of signaling pathways applied to drug discovery, may provide a new path to approach this question [2, 6, 7]. Mechanistic understanding at the network level offers integrated insights about the cellular responses to environmental changes and drug effects, yielding a significant understanding of the signaling cascades derived from decades of research in this field [8–10]. Mathematical modeling of signaling networks has been used to unravel signaling mechanisms and discover drug targets and disease mediators such as cell surface receptors or intracellular molecules by training those models to experimental in vitro measurements of key pathway components using inhibitors and activators [4, 11, 12]. Modeling-based studies may be the key to characterize the effect of drug combinations at the molecular level and allow us to predict both efficacy and reduction of off-target effects [5, 11, 13].

Multiple sclerosis (MS) is an autoimmune disease, in which the immune system is chronically activated and damages the central nervous system (CNS) [14]. The involvement of immune system deregulation in MS is shown by altered phenotype and activity of blood lymphocytes and monocytes [14], as well as by association with genetic polymorphisms of immune genes [15]. At present, there are fifteen Food and Drug Administration (FDA)-approved immunomodulatory drugs, and many others are in late-stage clinical development. Most of these control the inflammatory activity in patients with MS to a certain degree, though with known unwanted effects at the signaling level. Furthermore, many unmet medical needs remain in the attempt of achieving control of the disease including more effective therapies, a good safety profile, and neuroprotective or regenerative treatments. Drug combinations are considered as a promising strategy to overcome some of these limitations, and in cancer combination therapies, they are well established [16]. However, predicting which patients would benefit the most from a certain combination therapy remains as an unresolved challenge [17–20].

**Fig. 1:**
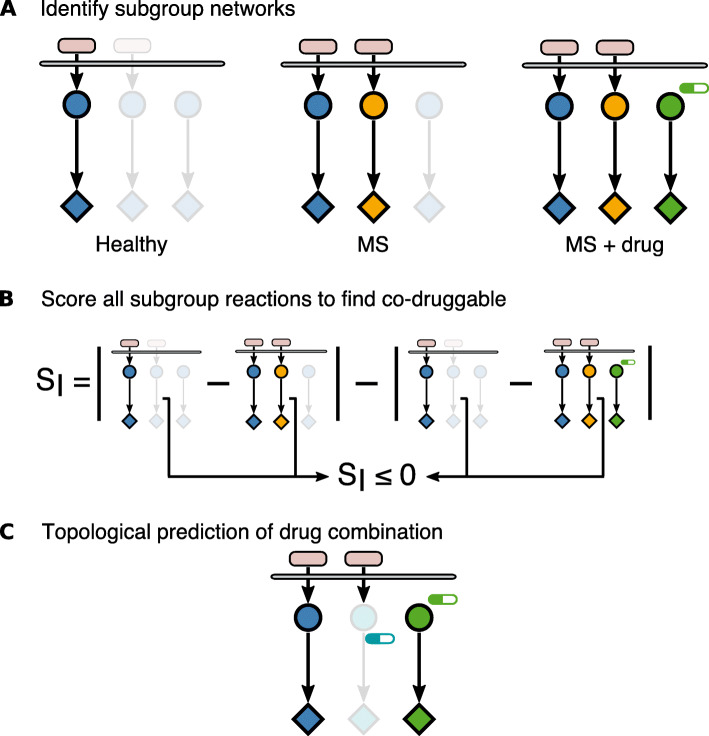
Topological modeling approach of signaling pathways for prediction of combination therapy. **a** Identification of subgroup networks. A model characterizing signaling activity (upstream kinase (circle) that regulates a response, e.g., innate immunity, survival (diamond)) in response to a stimulus (oval) was calculated for each donor based on the experimentally acquired dataset. Next, the donor-specific models are merged for all donors belonging to the same subgroup (left panel blue: healthy controls; middle panel orange: untreated MS; right panel green: treated MS). **b** Scoring subgroup interactions to find co-druggable network interactions. The score is calculated to identify interactions that differ from healthy-like signaling activity in spite of drug treatment (see the “Methods” section). **c** Topological prediction of drug combination. A topology-based graph search allows identifying secondary treatments that could target and revert signaling of co-druggable interactions to a healthy-like activity state

In this study, we present a systems medicine approach aimed to (i) characterize the signaling pathways in primary immune cells obtained from the blood of MS patients and healthy controls and (ii) predict new combination therapies based on the differences in signaling networks between treated MS patients and controls (Fig. 1). To this end, we assembled a literature- and database-based prior knowledge signaling network (PKN) [21], which includes the pathways involved in immune and MS signaling, as well as their crosstalk and known therapeutic targets. Based on their ability to model large networks with a low number of parameters, logic models have been used to unravel the network topology driving disease and response to therapy [22–25]. Here, we established a Boolean logic model from the signaling network and trained it with measurements of kinase de/phosphorylation as a proxy of signal propagation upon perturbation with ligands and drugs in the peripheral blood mononuclear cells (PBMCs) from MS patients and healthy controls. We determined disease- and therapy-specific logic models that characterize the signaling networks for approved treatments (interferon-beta (IFNβ), glatiramer acetate (GA), natalizumab (NTZ), fingolimod (FTY), and the experimental drug epigallocatechin gallate (EGCG)). We hypothesized that the signaling interactions that the drugs failed to revert in the ex vivo assays to a healthy-like activity level may be candidates to be targeted by a second drug and hence lead to a personalized combination therapy. To identify those interactions, we developed a score of co-druggability of signaling interactions according to quantitative differences in network topology among healthy controls and untreated and treated MS patients (Fig. 1 and Table 1).

**Table 1:** Overview of the identification of co-druggable interactions: co-druggability score examples and their interpretation

Healthy	MS	Treated	Score	Treatment Affecting?	Healthy-like signaling after treatment	Interpretation
0	0	0	0	**NO**	**YES**	Healthy-like signaling: No combination therapy needed
0	0	1	-1	**YES**	**NO**	Un-healthy signaling. Single treatment is affecting: **Co-druggable**
0	1	0	1	**YES**	**YES**	Healthy-like signaling. Effective single treatment: No combination therapy needed
0	1	1	0	**NO**	**NO**	Un-healthy signaling. Single treatment not affecting: **Co-druggable**
1	0	0	0	**NO**	**NO**	Un-healthy signaling. Single treatment not affecting: **Co-druggable**
1	0	1	1	**YES**	**YES**	Healthy-like signaling. Effective single treatment: No combination therapy needed
1	1	0	-1	**YES**	**NO**	Un-healthy like signaling. Single treatment is affecting: **Co-druggable**
1	1	1	0	**NO**	**YES**	Healthy-like signaling. No combination therapy needed

Co-druggable: interactions where treatment with the drug alone yielded signaling activity different to that of the healthy-like state. Columns 1–3: Signaling activity of a given interaction as assessed by modeling in healthy, untreated MS and treated MS. Column 4: Co-druggability score calculated based on differences between columns 1-3. Column 5: Difference present between treated and MS signaling. Column 6: Absence of a difference in interaction between healthy and treated signaling. Column 7: Combination of columns 4, 5 and 6 to identify interactions deregulated (i) by the disease and not reverted to healthy state by treatment or (ii) by an off-target signaling effect by the primary drug (see “Methods”)

Using this network-based approach, we predicted several combination therapies, in particular of TAK1 with all five studied MS drugs. We validated a highly scoring combination therapy in the animal model of MS experimental autoimmune encephalomyelitis (EAE). The modeling approach shown here can be used for designing combination therapies for other complex diseases as well as for developing personalized therapies.

## Methods

### Subjects and clinical cohorts

We recruited 255 subjects including 195 patients with MS and 60 healthy controls in a multi-centric study in four MS centers (Hospital Clinic Barcelona – IDIBAPS (n=69), Karolinska Institute (n=64), University of Zurich (n=40), and Charité University (n=82)). Healthy donors, untreated patients and patients treated with IFNB, GA, NTZ, and FTY were described before in an accompanying study [26], whereas the participants in a trial testing epigallocatechin-gallate (EGCG) at Charité University were described in [27]. Characteristics of the clinical cohorts: mean age: 43.1+11.3 years; disease duration: 8.7+7.7 years; median Expanded Disability Status Scale (EDSS): 2.0 (0–6.0); disease subtype: 24 clinically isolated syndrome (CIS), 129 relapsing-remitting MS (RRMS), 6 secondary-progressive MS (SPMS), and 36 primary-progressive MS (PPMS); untreated: 93 and 60 healthy controls (Table 2). In order to account for sex and age, healthy controls were matched to the RRMS subgroup, i.e., the most prevalent subpopulation which is also the MS subtype most frequently treated, a requirement to enable our goal to characterize signaling deregulation upon treatment. After quality control of the phosphoproteomic dataset, the number of subjects was reduced to 169 (129 MS patients and 40 healthy controls), see Additional file 1: Supplementary methods.

**Table 2:** Demographic and clinical variables of MS patients and healthy controls (HC)

	MS ***n***=195	HC ***n***=60
**Sex (M/F)**	66/129	21/39
**Age**	43.1±11.3	39.9±8.5
**Disease duration (months)**	104.9+93.2	--
**Age at onset**	34.5+10.3	--
**EDSS**	2 (0-6.0)	--
**Disease subtype**		
**CIS**	24	--
**RRMS**	129	--
**SPMS**	6	--
**PPMS**	36	--
**Treatments**		
**DMD**		
**FNb**	36	--
**GA**	18	--
**NTZ**	22	--
**FTY**	20	--
**Experimental drug**		
**EGCG**	6	--
**Untreated**		
**Untreated**	93	--

*M* male, *F* female, *EDSS* Expanded Disability Status Scale, *CIS* clinically isolated syndrome, *RRMS* relapsing-remitting MS, *SPMS* secondary-progressive MS, *PPMS* primary-progressive MS, *DMD* disease-modifying drug, *IFNb* interferon beta, *GA* glatiramer acetate, *NTZ* natalizumab, *FTY* fingolimod, *EGCG* epigallocatechin-gallate

### Samples and processing

A unified standard operating procedure for PBMC isolation, stimulation, and lysis, as well as sample storing and shipping was developed along with a kit (plates) with reagents and buffers that were produced in a single facility (ProtAtOnce) and shipped to all participating centers (see Additional file 1: Supplementary methods for details). The reagents were prepared from a single batch, and plates were prepared from a single batch for each stimulus. Quality controls were carried out to ensure that the reagents remain stable for 3 months.

#### *xMAP assays*

XMAP assays were developed by ProtAtOnce (Athens, Greece) and were standardized to minimize error. We optimized assays from a list of 70 candidates (see Additional file 1: Supplementary methods) and obtained a final list of 17 phosphoproteins which display a good signal to noise ratio to be measured in the in vitro assays: AKT1, CREB1, FAK1, GSK3A, HSPB1, IKBA, JUN, MK03, MK12, MP2K1, PTN11, STAT1, STAT3, STAT5A, STAT6, TF65, and WNK1 (Additional file 2: Tables S1 and S2). We used a set of 20 stimuli, which included pro-inflammatory or pro-oxidant stimuli (Anti-CD3, concanavaline A (conA), IFNG, IL1A, IL6, LPS, NaCl, PolyIC, TNFα), immunomodulatory stimuli (S1P, vitD3) neuroprotectants or anti-oxidants (BDNF, EGCG, INS, and BN201), disease-modifying drugs from MS (DMF, FTY, Teriflunomide, IFNβ1a (Rebif®)), CNS-damaging oxidative stress H_2_O_2_, and a culture media as control (Additional file 2: Table S3). Samples were collected at baseline (time 0) and after 5 and 25 min.

#### Data normalization

After signal reading, data was normalized. To allow logic modeling, data was normalized between 0 and 1, extending via stringent statistics the normalization strategy presented in [21] (see Additional file 1: Supplementary methods). The maximum between 5 and 25 measurements was selected to allow capturing signal transduction including late effects of network motifs such as negative feedback loops.

#### Model generation

A model describing core immune signaling was established based on Saez-Rodriguez et al [28]. Next, the model was extended to further immune and MS-related pathways such as interferon response, B and T cell receptor signaling, cellular survival and apoptosis, inflammation, lipid signaling, innate immunity, and multi-drug response (MDR) genes from the state-of-the-art databases such as KEGG [29], ScienceSignaling [30], and Wikipathways [31]. To allow the inclusion of MS drugs, the drug targets were included from ChEMBL [32] and Uniprot [33] in pathways as explained above via the references detailed in Additional file 2: Tables S4 and S5. Manual curation to prioritize interactions that were found in immune or human cells was used whenever possible. The global network featured (167 nodes and 294 interactions). The identifiable components, i.e., those that led to unique solutions using the optimization algorithm, were assessed as described in [21], yielding a so-called preprocessed network of 71 nodes and 168 interactions. To reduce model complexity to a degree that could be solved by model optimization, AND gates were added based on publicly available knowledge on protein complexes at EBI’s complex portal [34] (https://www.ebi.ac.uk/intact/complex) (see Additional file 1: Supplementary methods). Next, a personalized model for each donor was generated using individual datasets as described in the “Model optimization” section.

#### Model optimization

For each patient, 10 completed optimization runs were performed with a genetic algorithm and assessed as shown in Additional file 3: Figure S1 (see Additional file 1: Supplementary methods).

#### Network topology-based prediction of combination therapy

We calculated the co-druggability score as described in Fig. 1 with *S* indicating the signaling activity status of each interaction:

$$ \mid {\mathrm{S}}_{Healthy}-{S}_{MS}\mid -\mid {\mathrm{S}}_{Healthy}-{S}_{Treatment}\mid $$

were *healthy* refers to the healthy subgroup model, *MS* relates to the untreated MS subgroup model, and *treatment* denotes one of the five approved drug-treated MS subgroup models. Therefore, interactions with a negative co-druggability score indicated a treatment effect that produced signaling activity more different from that found in healthy donors than without treatment and were selected as co-druggable. A co-druggability score of 0 indicated that there was no effect due to drug treatment. From those cases, the interactions in which signaling activity was different between healthy donors and treated patients were also selected as co-druggable, i.e., those where the drug alone failed to revert signaling to a healthy state.

$$ \mid {\mathrm{S}}_{Healthy}-{S}_{MS}\mid -\mid {\mathrm{S}}_{Healthy}-{S}_{Treatment}\mid \kern0.5em \le 0 $$

Thus, we used the algorithm described in Fig. 1 to define the co-druggability of all interactions within each treatment group after filtering for active signaling. To ensure that interactions with positive scores close to zero were also captured by our method (interactions with a similar signaling activity between drug treatment and MS untreated), we defined a lower quartile threshold around zero and collapsed almost zero scores into zero. Further, we required co-druggable interactions to be significantly (p<0.05) different between healthy and drug signaling by using the upper quartile as the difference threshold. Applying both filters together allowed us to identify co-druggable interactions that were deregulated by the disease as well as the unwanted signaling effect by the primary drug (Additional file 3: Figure S2).

#### Experimental autoimmune encephalomyelitis

Female C57BL/6 mice from Harlan (8–12 weeks old) were immunized subcutaneously in both hind pads with 300 μg of a myelin oligodendrocyte glycoprotein (MOG_35-55_, Spikem, Florence, Italy) emulsified with 50 μg of *Mycobacterium tuberculosis* (H37Ra strain; Difco, Detroit, MI, US) in incomplete Freund’s adjuvant as described previously [35]. Mice were injected intraperitoneally with *Pertussis toxin* (500 ng; Sigma, US) at the time of immunization and 2 days later. Animals were weighed and inspected for clinical signs of disease on a daily basis by an observer blind to the treatments. The severity of EAE was assessed on the following scale: 0= normal; 0.5= mild limp tail; 1= limp tail; 2= mild paraparesis of the hind limbs, unsteady gait; 3= moderate paraparesis, voluntary movements still possible; 4= paraplegia or tetraparesis; 5= moribund state; 6= death. Animals (10 mice per arm) were randomized to their treatment once they have reached a clinical score > 1 point. Clinical assessment was performed by a blinded evaluator to the treatment group. Comparison between groups was performed using the Mann-Whitney test with p value cut-off at 0.05.

#### Statistical confirmation of patient subgroup models

To test whether the signaling models found for each patient subgroup were reflected in the experimental phospho-levels, we followed a three-stage strategy. First, we identified stimulus-responding readouts in our experimental dataset by assessing for each stimulus-readout combination if the fold changes (before non-linear normalization) of a given readout deviated significantly from 0 for all donors within a single patient subgroup using a one-sample Wilcoxon test. Multiple testing correction was performed using the Benjamini-Hochberg strategy, yielding a p value for each stimulus-readout combination within a group. Secondly, we determined which stimulus-readout pairs were either in accordance with the model of signaling or not, by confirming if a phospho-readout could be reached from the respective stimulus in the signaling model for the different subgroups (Additional file 3: Figure S3A-E). Finally, using Fisher’s exact test, we calculated whether the stimulus-readout pairs found to be significant (using p<0.05 as cutoff) in the first stage were enriched for those supported by the model (determined in the second stage) compared to those not supported (Additional file 3: Figure S3F).

## Results

### Multiplexed phosphoproteomic analyses in PBMCs from MS patients and controls

To characterize the signaling networks involved in MS, we created a Prior Knowledge Network (PKN) of biochemical interactions (kinase phosphorylation) reported to be involved in immune signaling associated with MS based on omics and functional studies, as well as those pathway interactions targeted by MS drugs [17]. Our network includes pathways such as interferon response, B and T cell receptor signaling, cellular survival and apoptosis, inflammation, lipid signaling, innate immunity, and multi-drug response (MDR) genes (Additional file 3: Figure S4). To achieve this, we searched in the state-of-the-art databases for interactions that were reported in highly specific assays and prioritized experiments with human and PBMC cells (see the “Methods” section). Further, targets of MS drugs were included in the network via their crosstalk with immune pathways. The PKN featured 167 signaling components (Additional file 2: Tables S5) and 294 interactions (Additional file 2: table S4).

Subsequently, we developed a multiplex xMAP phosphoprotein panel as a proxy of protein activation. The phosphosites in the panel were selected to maximize the coverage of the MS-specific network. The stimuli were selected according to their regulation of the pathways included in the PKN as detailed above and referenced in Additional file 2: Tables S4 and S5. The panel, which combined previously and newly developed assays, was then optimized to maximize accuracy, reproducibility, and network coverage (see Additional file 2: Table S1 and Additional file 1: Supplementary methods) [36]. After optimization, we selected a set of 17 phosphoproteins with adequate signal to noise ratio that were used for the in vitro assays: AKT1, CREB1, FAK1, GSK3A, HSPB1, IKBA, JUN, MK12, MK03, MP2K1, PTN11, STAT1, STAT3, STAT5, STAT6, TF65, and WNK1 (Additional file 2: Table S2). PBMCs were cultured in the presence of different sets of stimuli such as lectins, endotoxins, immunostimulants, cytokines, and drugs (Additional file 2: Table S3).

To estimate the representativeness of our selected phospho-signals, we calculated closeness centrality, a metric which measures the efficiency of a node in spreading information through a graph, for all nodes in the immune- and MS-specific signaling network presented here. No signal was found to be central to the whole network, and the nodes we selected and measured as signals were found to be the most central nodes in the B cell, T cell, inflammation, IFNb, cellular survival, apoptosis, and innate immunity pathways (Additional file 3: Figure S5), thereby supporting that they were indeed representative of the pathways we sought to study.

We extracted PBMCs from 195 MS patients (mean age: 43.1+11.3 years; disease duration: 8.7+7.7 years; median Expanded Disability Status Scale (EDSS): 2.0 (0–6.0); subtype: 24 CIS, 129 RRMS, 6 Secondary-Progressive MS (SPMS), and 36 PPMS; untreated: 93) and 60 healthy controls (Table 2). From those 255 donors, 180 were selected and PBMCs were analyzed before stimulation and at 5 and 25 min after stimulation. This yielded a dataset consisting of three measurements for 17 phosphoproteins upon 20 stimuli, to a total of 183,600 experimental measurements. For subsequent modeling, the two-time points after baseline (5 and 25 min) were collapsed into a single activation signature to define whether a given phosphoprotein was activated or inhibited (see details in the “Methods” section). Finally, we applied stringent quality control analyses of the dataset consisting of positive and negative controls, reproducibility tests, and tests for artifacts in the distribution of the samples (see Additional file 1: Supplementary methods), which led to the removal of 2028 data points and 11 patients to generate the final dataset for a total of 169 subjects. To reduce the complexity of the model to those pathways that can be determined based on the experimental coverage, identifiability analysis was performed as described in [21]. After identifiability analysis, the PKN (shown in Additional file 3: Figure S4) was reduced to 71 proteins and immune modulators and 168 interactions.

### Logic modeling enables characterization of signaling networks in healthy donors and MS patients

**Fig. 2:**
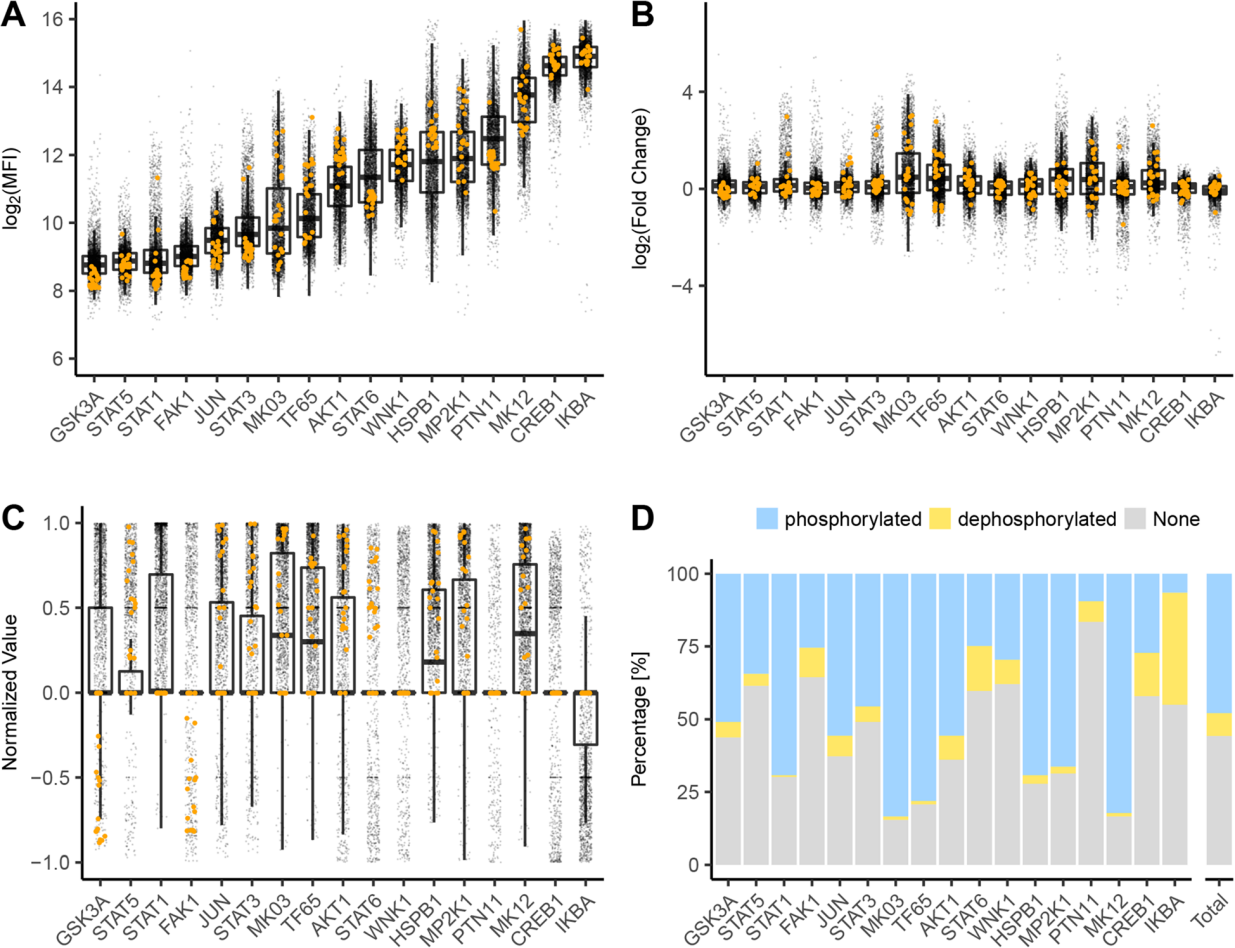
Phosphoproteomic measurement and normalization pipeline. **a** xMAP mean fluorescence intensity (MFI) log values of the 17 analyzed phosphoproteins, **b** fold change distribution, **c** non-linearly normalized values (see the “Methods” section). Orange measurements **a**–**c**: values of the same patient to allow visualization of the changes across data transformation. **d** Percentage of patients, for which each phosphoprotein was classified as phosphorylated, dephosphorylated, or non-significant after statistical testing

Next, we sought to train the generic PKN to these phosphoproteomic measurements in a donor-specific manner to identify each donor’s active pathways. To train the same Boolean logic model to the data of each of the 169 subjects, the phosphoproteomic datasets were transformed (Fig. 2a). To enable comparison of the data to the binary output of the Boolean model, we established a stringent normalization pipeline combining (i) a non-linear transformation to normalize the measurements to continuous values between 0 and 1 while penalizing the outliers [21] with (ii) a statistical filter that removed data-points belonging to proteins which, upon perturbation, were not found to be significantly phosphorylated or dephosphorylated. Following this strategy to identify two significant states from continuous phosphorylation measurements featuring a three-state scenario of phosphorylation, dephosphorylation, or unchanged levels, this procedure enables us to train a Boolean (binary) model (Additional file 1: Supplementary methods, “Data normalization for logic modeling” and Additional file 3: Figure S6). Figure 2b shows the log fold change of each phospho-profile with respect to the control, Fig. 2c the dataset after the non-linear normalization, and Fig. 2d displays the proteins found to be significantly dephosphorylated or phosphorylated. Using this normalized dataset, we aimed to identify the active specific signaling network for each donor.

**Fig. 3:**
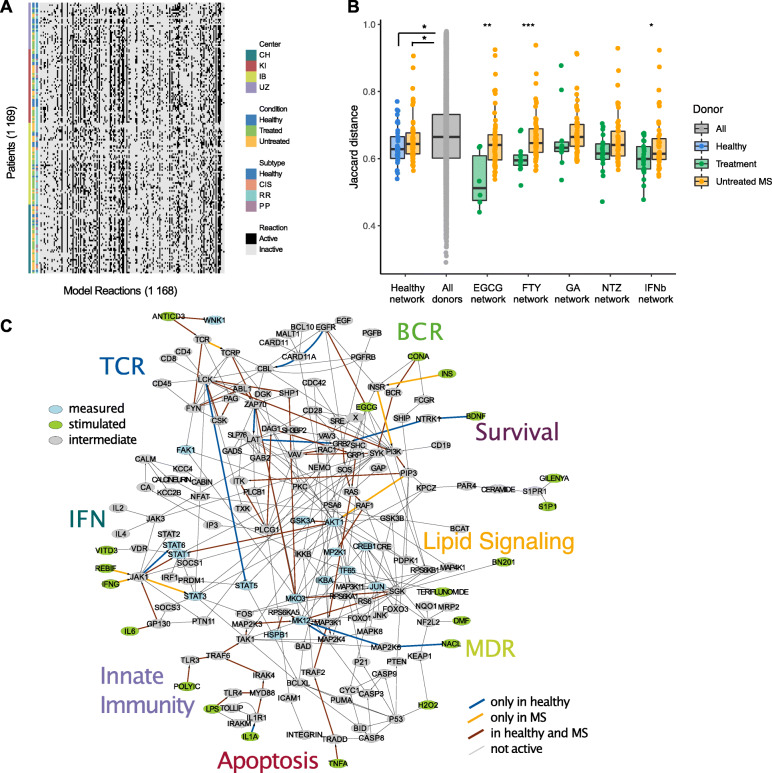
Logic modeling identifies donor-specific signaling networks and reveals MS-specific signaling pathways. **a** Signaling network found by modeling for each donor, visualized as a heatmap. Rows: Single donor network. Columns: Signaling activity determined for each interaction by calibrating the PKN shown in Additional file 3: Figure S4 after removing the unidentifiable interactions using the phosphoproteomics dataset of each donor. **b** After networks were merged by subgroup, the Jaccard distance was used to assess similarity from all donors within each group (selected donors in group legend) to their mean subgroup network (network in X axis) and compare it to the similarity from MS patients to the same group network. Healthy donors (blue) were more similar to the mean healthy network than untreated MS patients (orange). In turn, the distance from both groups of donors to that of the combined signaling activity in all donors (grey) was statistically significant. Distance from treated donors (green) to their mean subgroup network was largely reduced when compared to distance from untreated donors to the treatment’s network, suggesting a strong effect of treatment homogenizing within group signaling. **c** Differentially activated pathways (see Additional file 1: Supplementary methods) between healthy controls (HC) and untreated MS patients (MS). The models previously calculated for each donor were merged to reveal the common active pathways for controls (blue), untreated MS patients (orange), and both (brown). Gray: Inactive interactions from the MS, immune- and treatment-related network (Additional file 3: Figure S4)

To this end, we fitted a general logic model derived from the PKN [21] to each donor dataset, which resulted in a compendium of 169 models. Model optimization was performed with CellNOpt, a tool that selects the logic model that best matches the data while penalizing model size [37]. To increase the robustness of each model, optimization was performed 10 times, and for each donor, we selected the best solution as well as all models with a data-to-simulation mismatch within a given relative tolerance reflecting that, due to lack of identifiability and technical noise, different models are feasible [21] (see the “Methods” for details). We then built a final model for each patient, defined by the median value of each interaction across all solutions within the relative tolerance (or borders of tolerance) of the best solution (Fig. 3a). To assess the validity and robustness of our modeling approach, we confirmed the absence of bias due to treatment, center, disease subtype, medical condition, and technical aspects such as model size and merging strategy (Additional file 3: Figure S1).

To study the effect of MS and MS treatment on signal transduction, we first merged the individual donor models within each group by calculating the mean activity over each reaction, which yielded a single network per group. Next, we calculated the distance between each patient model and the corresponding group mean network using the Jaccard distance, a metric used to assess the number of interactions that are dissimilar in two networks [38]. Finally, as a comparison, we calculated for each group the distances between the mean group network and the networks of all untreated MS patients (Fig. 3b). We found that healthy donors were slightly more similar (median Jaccard distance = 0.628) than MS untreated patients (median Jaccard distance = 0.644) to the mean healthy signaling network. We then calculated pairwise distances among all donors, and used them as a background similarity for comparison. We found the dissimilarity among healthy donors to be significantly lower than that of the background (Wilcoxon test’s p value = 0.0215). The dissimilarity in untreated patients was also lower than the background’s (p value = 0.0318). In addition, drug treatment exhibited a strong effect on signaling, which seemed to be homogenized within each group with dissimilarities lower than that of healthy and untreated donors (median Jaccard distance to each group’s mean network: EGCG = 0.512, untreated MS = 0.641; FTY = 0.595, untreated MS = 0.646; GA = 0.632, untreated MS = 0.665; NTZ = 0.615, untreated MS = 0.641; IFN = 0.599, untreated MS = 0.615). The differences were found to be significant in three of the treatments (Wicoxon’s test p value EGCG = 0.00676, FTY = 0.000418, IFNb = 0.0462), further supporting the strong effect of drug treatment on signaling. Altogether, these results supported that merging models by subgroups of donors yields biologically meaningful signaling networks for each group.

Next, we sought to characterize the specific network architectures of MS, as well as those of MS treatments. The differentially activated interactions between healthy donors and untreated MS patients uncovered signaling pathways deregulated in MS (Additional file 1: Supplementary methods and Fig. 3c). Our analysis revealed the activation of several pathways in PBMCs after stimulation both in patients and controls, ranging from cell survival and proliferation to TCR, innate immunity, and pro-inflammatory response pathways (e.g., TCR - CSK - LCK, JAK1 - STAT1, TLR4/IL1R1 - MYD88 - IRAK4 - TRAF6 - TAK1, TNFα - TRADD - TRAF2 - MAP3K1, SGK - MK03, or RS6 - SGK, RAF1 - MP2K1). Further, increased activity was found in pathways such as INSR - PI3K - PIP3 - AKT1 and JAK1 - STAT3 in MS patients, while BDNF - NTRK1 - GRB2 was found to be activated in healthy donors.

These findings indicated that our method was able to identify previously described pathways in the setting of ex vivo analysis of human PBMCs [17], as examined in detail for the individual pathways in the discussion. Untreated MS patients, when compared to healthy controls, showed an increase in the activation of the NFkβ pathway (TAK1-IKKB), the activation of the cell prosurvival PI3K pathway (SLP76-AKT1), or the activation of interferon/cytokines pathways (JAK1-STAT3). Moreover, patients showed a decrease, compared to healthy controls, in the activation of TCR/IL-2 pathway (LCK-STAT1) as well as in the effect of trophic factors signaling on ubiquitination system (EGFR-CBL). These results characterize, at the mechanistic and quantitative level, the differential activation of the immune pathways in MS and were supported by the statistical analysis of the phosphorylation of individual kinases in an accompanying study [26]). In particular, the accompanying study found MP2K1, STAT1, STAT3, TF65, and HSPB1 to be differentially phosphorylated (p<0.05 after correction for multiple hypothesis testing) in MS patients relative to the controls, confirming the differential activation of cell survival and proliferation (MAPK), and pro-inflammatory (STAT) pathways in immune cells.

### Quantitative differences found in signaling pathways under therapy

We then quantified differences in signaling between patients treated with MS-specific therapies, using the same procedure as for healthy donors versus untreated MS patients. We focused on the strongest signals, i.e., those in the upper quartile of the group mean (Additional file 3: Figure S2). This yielded a signaling network of active reactions characterizing each subgroup, uncovering the effect of each MS-treatment on signaling at the mechanistic level (Additional file 3: Figure S3).

Interestingly, the activation of TAK1, a key component involved in TGF-β, Toll-like receptor, B cell receptor, and NFkβ pathways, as well as response to inflammation was identified in all five networks inferred under treatment (Additional file 3: Figure S3 A-E). Additionally, its activity was persistently over-activated in terms of quantitative signaling activity by the currently approved MS drugs (Additional file 3: Figure S2). In the case of IFNβ- and GA-treated patients, the network revealed the activation of STAT1 and STAT3 by JAK1, whereas in the EGCG-, FTY-, and NTZ-specific networks, the activation of the JAK1-STAT pathway is regulated via STAT3, STAT6, and STAT1, respectively.

To validate that the therapy-specific pathways found were consistent with the experimental measurements of MS signatures, we quantified the degree to which the original phospho-measurements supported the signaling pathways predicted by modeling for each patient subgroup. The proteins differentially phosphorylated across all stimuli combinations were overrepresented in pathways found by model fitting (IFNβ p=1.2E−05; GA p=1.4E−06; FTY p=0.0004; NTZ p=0.006; Fisher’s exact test) except for EGCG, which was limited by the small sample size (Additional file 3: Figure S3F and methods).

### Network topology-based prediction of targeted combination therapy

**Fig. 4:**
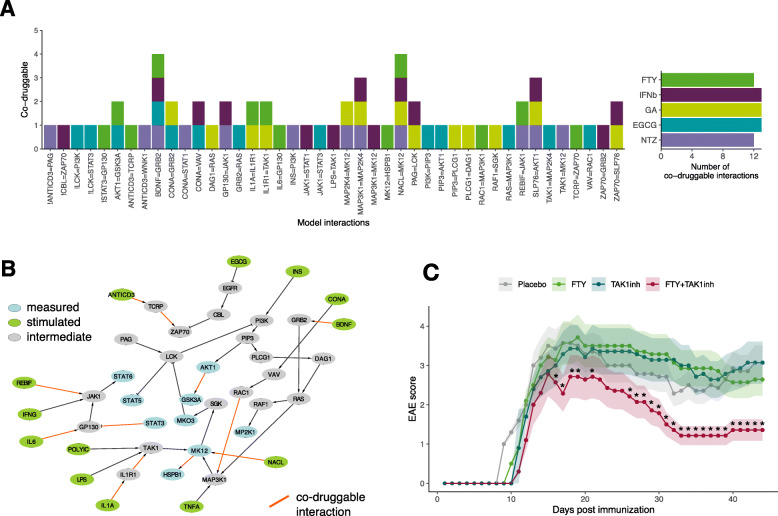
Combination therapies predicted and in vivo validation. **a** All predicted co-druggable interactions of the MS drugs models. Based on the subgroup models, the co-druggability of all 168 network interactions (X axis) was assessed using the co-druggability score, and those identified as co-druggable (see Fig. 1, Table 1 and main text) are shown. For each interaction (X-axis) the number of drugs (Y-axis) is shown, in which it was found to be co-druggable using the co-druggability criteria. **b** FTY network co-druggability: the case of FTY network co-druggability is shown as an example (red line: interactions predicted to be co-druggable). **c** In vivo validation of the combination FTY+TAK1-inhibitor in the EAE model. The graph shows the mean and the standard error of the clinical score for each group (*n*=7). Animals started treatment after disease onset (clinical score >1.0) and were randomized to each treatment and rated in a blinded manner. Stars show days significantly different from the same day with placebo

Our main goal was to use the subgroup networks found to predict novel combination therapies. To this aim, we defined a therapeutic goal to revert the signaling network of patients with MS to a healthy-like activity. We introduced a restriction to our method: the combinations we sought included one approved MS drug which, in spite of its known efficacy in MS, yielded a signaling activity that differed from the healthy controls as identified by our topological modeling approach. Therefore, we aimed to identify which kinase interactions within the network needed its signaling activity to be reverted to the healthy-like state when treated with the ongoing therapy. We hypothesized that co-druggable interactions, i.e., those that a given MS therapy failed to revert to a healthy-like activity level, should be the model interactions with a signaling value more distant between healthy control and MS drug models than between healthy control and untreated MS models (Fig. 1, Table 1 and see the “Methods” section). This yielded a list of interactions with their corresponding score quantifying co-druggability potential (Additional file 2: Table S6 and Additional file 3: Figure S2). To identify the co-druggable interactions, we selected those with a non-positive score as described above and filtered for additional conditions (see the “Methods” section), thereby identifying both the interactions whose signaling activity was different from healthy because of deregulation by the disease as well as because of off-target primary drug effect (Fig. 4a). The last step of our approach was to predict the stimulus that would revert to healthy-like signaling activity of those interactions identified as co-druggable. Therefore, the co-druggable interactions were mapped onto the signaling network assessed for each drug subgroup. As an example, Fig. 4b shows the co-druggable interactions under FTY treatment mapped onto the FTY network. Mapping the co-druggability for each interaction allowed us to predict combination therapies based on each treatment’s signaling network topology. Finally, we employed a graph search approach to identify the in vitro stimulus used in our study that activated interactions found to be co-druggable with each drug. In other words, we found the experimental readouts that could be measured and reached from an in vitro stimulus via an interaction found to be co-druggable in vivo (Additional file 2: Table S7). It is important to note that our approach enabled the prediction of combinations of approved MS therapies which yielded a signaling activity distant from healthy controls with drugs that stimulated or inhibited signaling. We statistically confirmed that the signaling models found for each patient subgroup were reflected in the experimental phospho-levels (see statistical confirmation in the “Methods” section and Additional file 3: Figure S3).

### Validation of a predicted combination therapy in the animal model of MS

Next, we validated in vivo one of our predictions. Among all predictions (Additional file 2: Table S7), we chose to validate the combination of FTY with a TAK1 inhibitor based on (i) the striking signaling homogeneity across FTY models (as quantified in Fig. 3b), (ii) the finding that TAK1 modulation of HSPB1 via MK12 is largely deregulated (Additional file 3: Figure S2, panel FTY, interaction MK12 - HSPB1), and (iii) the fact that TAK1 is active and co-druggable in all 5 networks under treatment with in vivo drugs, specifically TAK1 - MAP2K4 for ECGC, IL1R1 - TAK1 for FTY, IL1R1 - TAK1 for GA, LPS - TAK1 for IFNβ, and TAK1 - MK12 for NTZ (Additional file 2: Table S6).

To obtain an in vivo proof-of-concept of the efficacy of the combination therapies proposed, we sought to assess whether the combination therapies improved the clinical course of the animal model of MS (EAE in the C57BL6 mice immunized with the MOG_35-55_ peptide). Mice were randomized after disease onset (after they reached a clinical score >1.0), either to placebo (saline), each drug alone, or the combination of FTY with the TAK1 inhibitor (5Z-7-oxozeaenol). Doses were selected from previous dose-efficacy studies [39] and [40] in the EAE model, whereby the next lower dose without efficacy below the efficacious one was selected. We found that the combination FTY+TAK1-inhibitor ameliorated the clinical course of EAE compared to placebo, TAK1-inhibitor, or FTY alone (Mann-Whitney test; *p*<0.05) (Fig. 4c). Therefore, our method was able to identify combinations of drugs targeting different pathways that achieved higher efficacy than single therapy.

## Discussion

In this study, we have built donor-specific dynamic logic models and developed a network-based approach to (i) characterize signaling deregulation in MS and several of its treatments and (ii) predict new targets for combination therapy. Although highly effective therapies have already been developed for MS, improving their efficacy further, particularly for progressive MS, remains an important unmet medical need [41]. Higher efficacy is desirable for progressive MS, in which currently approved drugs (Ocrelizumab and Siponimod) show approximately 20% amelioration in primary and secondary progressive MS respectively compared to placebo. Even in the case of relapsing-remitting MS, not all patients reach complete control of the disease (no evidence of disease activity (NEDA)). Recent studies have found that RRMS patients accumulate disability independently of relapses even if treated with low or high efficacy therapies as shown in the Ocrelizumab trials [42]. Whether the combinations that we identified with our approach would improve such outcome will require to be tested in clinical trials.

In addition to predicting combination therapies, the approach presented here allows insights into signaling deregulation in MS by comparing signaling networks in untreated MS patients with those of healthy controls. The comparison demonstrates enhanced pro-survival effects of the trophic factor signaling pathway (AKT1) and modulation of the interferon pathway (JAK1, STAT3). In line with the differences found here, aberrant STAT phosphorylation signaling in peripheral blood mononuclear cells from MS patients has been reported [43]. The NFkβ pathway has been reported to be overactivated in PBMCs from patients with MS [44, 45] as well as to contribute to the genetic susceptibility of the disease [46, 47]. Our analysis supports the systemic pro-inflammatory state of PBMCs in MS. The trophic factor pathway involving SLP76 and AKT was found active in patients under treatment with GA, NTZ, and IFNβ and has also been associated with MS [17] and MS susceptibility via CD6 gene [48, 49]. The involvement may reflect a pro-survival signaling state of T and B cells in the context of the pro-inflammatory microenvironment. Finally, we observed overactivation of the cytokine/interferon pathway (JAK1), which has previously been reported in MS [17]. Moreover, STAT3 has been confirmed as susceptibility gene for the disease [50], and its activation is impaired in response to IL-10 in MS patients [51], suggesting a defective response of regulatory Tr1 cells. Regarding the interactions that were decreased in MS patients compared to controls, PBMCs from patients with MS showed lowered inhibition of STAT5 by LCK suggesting impairment of the regulation of T/B cell signaling and IL-2 trophic effects [52] or cytotoxicity [53], as well as the regulation of the ubiquitination system modulated by CBL-B [54]. LCK is modulated by EVI5, and influences STAT5 in our analysis, both being susceptibility genes for MS [50]. The second inhibited interaction involved the MS susceptibility gene CBL-B [55], which regulates TCR and co-stimulatory signals and regulates immune tolerance through its ubiquitin E3-ligase activity. CBL-B expression is reduced in CD4 cells from MS patients and alters the signaling of the type I interferon pathway [56, 57]. CBL-B is activated by EGFR, leading to inhibition of several pathways by ubiquitination, including that of EGFR itself [58, 59]. In summary, our results are supported by multiple previous studies identifying deregulation of pathways in MS [17] and shows the activation of pro-inflammatory pathways and the inhibition of pathways related with immune tolerance.

Furthermore, the phosphoproteomic dataset designed for and used in this study was also used in an accompanying work [26] that performs a statistical comparison of phosphorylation of single kinases and their association to genetic susceptibility. The accompanying study found multiple differentially phosphorylated and associated kinases, which are crucial components of the pathways found deregulated here.

The combination therapies predicted with our topological modeling approach may contribute to reducing the treatment’s unwanted effects in two ways. First, our method enables the characterization of drug-specific effects on signaling. For example, the models revealed the activation of JAK1-STAT1 pathway [60, 61] in IFNβ-treated patients, the activation of AKT, PLC [62, 63] in patients treated with FTY, or the activation of MAPK, and NFkβ (via TAK1) [64–66] in NTZ-treated patients. By identifying models of MS drugs, our method found the pathways activated downstream of drug targets in our signaling network. Further, it identified interactions with other disease-associated pathways, which may not directly be involved in the signaling targeted by such a drug. Therefore, our modeling approach can be used to provide a wider context of the effects of a given drug on the functions of the target cells. Specifically, our co-druggability score identifies not only those interactions that are unhealthy-like due to MS deregulation but also those that change due to first line treatment, whose signaling can be reverted to healthy-like by a combination drug. Second, rational identification of drug combinations may allow us to use lower doses of approved drugs, thereby reducing the dose-related adverse events of each single drug, improving safety and tolerability while maintaining efficacy. As we show in this study, the latter aspect can be predicted from in vitro measurements and validated in animal models, as we did with the combination of FTY and a TAK1 inhibitor. In the specific case of FTY, its most common side effects are dose-dependent and include lymphopenia, transient bradycardia, increased rate of latent infections with HSV-1 and VZV, liver enzyme elevation, and a few others. Fingolimod acts by trapping lymphocytes in secondary lymphoid organs by functional antagonism of S1P receptors. However, its effects on cell signaling are poorly understood. Some of its adverse events may be prevented by reverting signaling to healthy-like in the TGF-β, Toll-like receptor, B cell receptor, NFkβ, or proinflammatory pathways of which TAK1 is a member. Further, in our validation, we made use of suboptimal doses and showed that the combination is effective in EAE mice as it is with Fingolimod at full dose, although using significantly smaller doses. Hence, safety can be reasonably expected to be improved. While these considerations regarding increased safety by drug combinations are reasonable, demonstrating them is likely more difficult and will require careful testing in patients. For instance, to date TAK1-inhibitors have not been and are not in clinical testing according to ClinicalTrials.gov for MS. However, TAK-1 inhibitor Takinib has been shown to broaden the therapeutic potential of anti-TNF approaches in cancer and autoimmune disease [67], and we anticipate that it is going to be developed for clinical testing. We hope that these insights can be used to reduce unwanted side effects and tailor treatment to patients.

Our study has several limitations. First, the analysis of signaling signatures is based on phosphoproteomics of mixed immune cells, i.e., PBMCs. While this allows us to measure key pathogenic MS features with a focus on immune mechanisms such as T cell and B cell activation, cell adhesion and migration, proinflammatory differentiation, and others in the multiple cell types that have been related to MS pathogenesis [68], it yields a signal that is the average of the signals in cell subtypes and hence can mask cell-type-specific responses. To avoid a signal averaged across PBMCs, one could filter specific immune cell subtypes such as CD4+, CD8+, B cells, and monocytes. Subsequently analyzing signaling abnormalities while accounting for differential contribution to disease susceptibility or response to therapy across cell subtypes may reveal new therapeutic targets. Alternatively, technologies to collect information at the single-cell level such as mass cytometry [69] would allow us to separate cells by type, but the cost is considerably higher, limiting the number of subjects. A more affordable alternative would be flow cytometry, which on the other hand can measure a lower number of analytes. Second, our current coverage using validated xMAP assays was restricted to 17 proteins. Although designed to maximize the coverage of immune- and MS-related pathways and confirmed as representative by topological analysis, these constitute a small subset of the signaling molecules and pathways that may be activated in immune cells. Some technologies such as mass spectrometry allow studying multiple phosphosites and at whole proteomic scale. However, the specific proteins measured are not chosen and cost per sample is much larger. Therefore, the number of patients, timepoints, and replicates would have to be reduced given our budget, making our study unfeasible. In those cases where upscaling the number of readouts is possible, robust quantitative phosphoproteomic assays covering thousands of phosphosites in hundreds of samples can be used for logic modeling [70]. This will allow us to significantly expand the scope of our models. Furthermore, other modifications such as ubiquitination can play an important role in signaling. Such data, if available, can be included in our models. Third, the Boolean logic approach does not describe processes biochemically and models processes as binary, hence missing subtle aspects of signal transduction [10]. One of the main goals of our study was to develop a modeling approach that can guide the rational development of combination therapies. Our signaling topology-based approach has the advantage of allowing prediction of combinations between drugs currently used in patients with MS and compounds that can stimulate signaling where those drugs yield a signaling activity distant from the healthy state. Many strategies have been developed to predict drug combinations [71]. Others have used phosphorylation data upon perturbation using statistical approaches [13], data-driven network inference [5, 11], or a combination of mechanistic and Bayesian network modeling [72]. Due to their simplicity, logic networks can model large networks and provide a useful framework to study drug combinations [23–25]. We used this logic framework to analyze a large dataset encompassing 183,600 data points derived from a newly recruited cohort of 169 donors. Our approach is a compromise between data availability, technical feasibility, and computational burden that sacrifices details to be able to capture a broad portion of the signaling machinery. Fourth, in this study, we have explored only some of the currently approved drugs for MS patients. And fifth, we have averaged individual patients’ networks to obtain the subgroup (drug) networks, which leads to losing individual variability, probably related to differential genetic susceptibility, or immune system activation.

In summary, we have built donor-specific dynamic logic models and developed a network-based approach to (i) characterize MS and treatment deregulation of signaling and (ii) predict new targets for combination therapy. This approach can be applied to other diseases.

## Conclusions

Here, we applied a modeling approach to characterize signaling activity both at the donor-specific and at the subgroup level using phosphoproteomic data from primary immune cells of MS patients and healthy donors. At the subgroup level, we identified models of the signaling network for healthy controls, untreated, and drug-treated MS patients. Our approach allowed us to characterize signaling deregulation in this complex and heterogeneous disease. Further, based on the data-driven models, we developed a network-based method to predict novel combination therapies for the treatment of MS. We hypothesized that the interactions in the subgroup models in response to single drugs that were not reverted to a healthy-like activity state can be co-drugged. Hence, we developed a co-druggability score identifying those interactions. Finally, we used that score on a newly developed strategy to predict combination therapies based on network topology. Thus, our algorithm identifies interactions within signaling networks that should be targeted to restore the network to a healthy-like state.

As validation, we tested in vivo the combination of one approved drug for MS, FTY, with an inhibitor of TAK1, a key component in the predicted signaling networks of all five studied in vivo drugs, which was found highly deregulated in FTY. This combination largely ameliorated the course of the animal model of MS with significantly higher efficacy than each treatment alone, providing in vivo proof-of-concept for our approach.

In conclusion, the approach developed here can be applied to other diseases with poorly understood pathogenesis and treatments which may produce severe side effects or only partially ameliorate the disease. We expect our approach will contribute to a better understanding of disease- and therapy-related signaling deregulation and aid in the development of combination therapies to restore healthy signaling.

## Supplementary Information

**Additional file 1.** Supplementary methods for phosphoproteomics dataset generation, data processing, statistical analyses, mathematical modeling and combination therapy prediction.

**Additional file 2: Supplementary Tables S1 to S7**.

**Additional file 3: Supplementary figures S1 to S6**.

## Data Availability

The data from clinical studies has been published by Kotelnikova et al. in [26], while the data from the EGCG clinical trial has been published by Bellmann-Strobl et al. in [27]. All data generated in this study are available on Github (http://github.com/saezlab/combiMS) [73], including all data transformations from xMAP raw measurements to modelling-transformed as shown in Fig. 2. All code and mathematical models can be found under the same Github repository.

## Abbreviations

MS: Multiple sclerosis
EGCG: Epigallocatechin gallate
TAK1: TGF-β activated kinase 1
TGF-β: Transforming growth factor beta-1 proprotein
CNS: Central nervous system
FDA: Food and Drug Administration
PKN: Prior Knowledge Signaling Network
PBMC: Peripheral blood mononuclear cell
IFNβ: Interferon-beta
GA: Glatiramer acetate
NTZ: Natalizumab
FTY: Fingolimod
EAE: Experimental autoimmune encephalomyelitis
RRMS: Relapsing-remitting MS
SPMS: Secondary-progressive MS
PPMS: Primary-progressive MS
CIS: Clinically isolated syndrome
EDSS: Expanded Disability Status Scale
