# Supplementary material for: Prediction of combination therapies based on topological modeling of the immune signaling network in multiple sclerosis
Source: Genome Med. 2021 Jul 16;13:117. doi: 10.1186/s13073-021-00925-8 (PMC8284018; doi:10.1186/s13073-021-00925-8)
Supplement: Supplementary file 2 — Additional file 2: Supplementary Tables S1 to S7. [file 13073_2021_925_MOESM2_ESM.pdf]

Additional file 2: Supplementary Tables S1-S7

Table S1. Selection of the phosphoproteins for the xMAP assays based on their involvement in pathways associated with MS or MS drugs, and assays performance. Phosphoproteins for the xMAP assays were selected based in a good signal to noise ratio to maximize network coverage and model identifiability

Protein	Phosphosite	Pathway relevance	Consensus	Multiplex-ability	SNR	Selection
AKT1	S473	HIGH	LOW	HIGH	LOW	PASS
CREB1	S133	HIGH	MEDIUM	HIGH	LOW	PASS
FAK1	Y397	MEDIUM		LOW	LOW	PASS
GSK3A	S21	MEDIUM	HIGH	MEDIUM		PASS
HSPB1	S78/S82	MEDIUM	HIGH		HIGH	PASS
IKBA	S32	HIGH	HIGH	HIGH	LOW	PASS
JUN	S63	HIGH		MEDIUM		PASS
MK03	T202/Y204	HIGH	MEDIUM	HIGH	MEDIUM	PASS
MK12	T180/Y182	HIGH	MEDIUM	HIGH		PASS
MP2K1	S217/S221	HIGH		MEDIUM	HIGH	PASS
PTN11	Y542	MEDIUM		LOW	HIGH	PASS
STAT1	Y701	HIGH	HIGH	HIGH		PASS
STAT3	Y705	HIGH	LOW	HIGH		PASS
STAT5	S694	HIGH		LOW		PASS
STAT6	Y641	HIGH		MEDIUM		PASS
TF65	S536	HIGH			HIGH	PASS
WNK1	T60	HIGH		HIGH	MEDIUM	PASS
EGFR	Y1068	HIGH		HIGH	HIGH	FAIL
GSK3B	S9	MEDIUM		HIGH	LOW	FAIL
KS6A1	S380	LOW	MEDIUM	HIGH	HIGH	FAIL
KS6B1	T389	LOW	HIGH	HIGH	LOW	FAIL
LAT	Y191	MEDIUM		LOW		FAIL
LCK	Y505	MEDIUM		MEDIUM		FAIL
MK09	T183/Y185	LOW	MEDIUM	HIGH	LOW	FAIL
MP2K6	S207/T211	LOW		LOW	LOW	FAIL
NRF2	S40	LOW		HIGH		FAIL
P53	S46	MEDIUM		HIGH	LOW	FAIL
PGFRB	Y751	LOW		HIGH		FAIL
RS6	S235/S236	MEDIUM		MEDIUM	LOW	FAIL
STAT2	Y690	HIGH		LOW		FAIL
VDR	S208	LOW		HIGH		FAIL
ZAP70	Y319	MEDIUM		MEDIUM		FAIL

Table S2. List of phosphoproteins used for the in vitro multiplex assays with PBMCs

Uniprot ID Name	Entrez-gene identifier	HGNC symbol	Uniprot Recommended Name	Uniprot Alternative Name	Gene Names	Antibody company catalog	Pathway	Biological role and Association with MS
**AKT1**	207	AKT1	RAC-alpha serine/threonine-protein kinase	Protein kinase B	AKT1, PKB, RAC	PAO P-AKT1-A01	PI3K/AKT/mTOR	key mediator of PI3K and mTOR signaling pathways, all involved in cell survival
**CREB1**	1385	CREB1	Cyclic AMP-responsive element-binding protein 1	-	CREB1	PAO: P-CREB1-A01	AP-1/MAPK	Participates in PI3K, MAPKinase pathways, promoting cell survival, neuronal activity, synaptic plasticity and expression of HLA molecules
**FAK1**	5747	PTK2	Focal adhesion kinase 1	Protein phosphatase 1 regulatory subunit 71	PTK2, FAK, FAK1	PAO: P-FAK1-A01	Integrin/src	key signaling mediator of integrin receptors and axon guidance as well as growth factor receptors (PI3K pathway)
**GSK3A**	2931	GSK3A	Glycogen synthase kinase-3 alpha	Serine/threonine-protein kinase GSK3A	GSK3A	PAO: P-GSK3A-A01	PI3K/AKT/mTOR	chemokine signaling pathway, being downstream AKT
**HSPB1**	3315	HSPB1	Heat shock protein beta-1	28 kDa heat shock protein	HSPB1, HSP27, HSP28	PAO: P-HSPB1-A01	p38/MAPK	Part of MAPKinase pathway, downstream p38, and promotes actin reorganization supporing cell migratio. Marked elevation in HSP27 levels during the relapse phase of MS.
**IKBA**	4792	NFKBIA	NF-kappa-B inhibitor alpha	I-kappa-B-alpha	NFKBIA, IKBA, MAD3, NFKBI	PAO: P-IKBA-A01	NFkB	NFkB signaling

**JUN**	3725	JUN	Transcription factor AP-1	Proto-oncogene c-Jun	JUN	PAO: P-JUN-A01	JNK/MAPK	JUN is part of the immediate early gene responses, being downstream JNK and mediating apoptosis
**MK03**	5595	MAPK3	Mitogen-activated protein kinase 3	ERK1	MAPK3, ERK1, PRKM3	PAO: P-MK03-A01	AP-1/MAPK	Member of the MAPKinase pathway, involved in proliferation, differentiation, and cell cycle progression
**MK12**	6300	MAPK12	Mitogen-activated protein kinase 12	MAP kinase p38 gamma	MAPK12, ERK6, SAPK3	PAO: P-MK12-A01	AP-1/MAPK	Member of the MAPKinase pathway, involved in proliferation, differentiation, and cell cycle progression
**MP2K1**	5604	MAP2K1	Dual specificity mitogen-activated protein kinase kinase 1	MEK1	MAP2K1, MEK1, PRKMK1	PAO: P-MP2K1-A01	AP-1/MAPK	Mediates signaling of PDGFR pathway, promoting cell survival
**PTPN11**	5781	PTPN11	Tyrosine-protein phosphatase non-receptor type 11	SHP2	PTPN11, PTP2C, SHPTP2	PAO: P-PTN11-A01	AP-1/MAPK	Member of the MAPKinase pathway, involved in proliferation, differentiation, cell cycle progression, cell transmigration, NK cytotxicity and axonal guidance
**STAT5A**	6776	STAT5A	Signal transducer and activator of transcription 5A	-	STAT5A, STAT5	PAO: P-STAT5-A01	JAK/STAT	Mediates IL-2, chemokines, NGR3-ErbB signaling and promoting cell survival. Upon IL-7 stimulation, MS patients experience stronger STAT5 activation in CD8-EM compared with HC.
**STAT1**	6772	STAT1	Signal transducer and activator of transcription 1-alpha/beta	Transcription factor ISGF-3 components p91/p84	STAT1	PAO: P-STAT1-A01	JAK/STAT	Mediates interferon gamma and beta signaling. Macrophages from MS patients displayed enhanced STAT1, STAT6 and NF-kB activity.

**STAT3**	6774	STAT3	Signal transducer and activator of transcription 3	Acute-phase response factor	STAT3, APRF	PAO: P-STAT3-A01	JAK/STAT	Mediates IL-6, IL-10, neurocytokines (LIF) signaling. STAT3 is required for IL-17 production by Th17.
**STAT6**	6778	STAT6	Signal transducer and activator of transcription 6	IL-4 Stat	STAT6	PAO: P-STAT6-A01	JAK/STAT	JAK-STAT pathway mediates Il-4 signaling. PBMCs from MS patients have significantly elevated constitutive phosphorylation of STAT6 compared to PBMCs from normal subjects.
**TF65**	5970	RELA	Transcription factor p65	Nuclear factor NF-kappa-B p65 subunit	RELA, NFKB3	PAO: P-TF65-A01	NFkB	Key member of NfKB pathway, meadiating inflammatory signals and cell survival. Macrophages from MS patients displayed enhanced STAT6, STAT1 and NF-kB activity.
**WNK1**	65125	WNK1	Serine/threonine-protein kinase WNK1	Erythrocyte 65 kDa protein	WNK1, HSN2, KDP, KIAA0344, PRKWNK1	PAO: P-WNK1-A01	ERK5/MAPK	EGF pathway and participate in the regulation of ion homeostasis and iron uptake

Table S3. Stimuli used for the in vitro multiplex assays with PBMCs

Stimulus	Full Name	pathway	cat number	Vendor	stock conc	units	target conc	units
antiCD3	Anti-Human CD3	TCR	16-0037-85	eBioscience	1	mg/ml	5000	ng/ml
BDNF	Brain-Derived Neurotrophic Factor human	TrkB/p75	B3795-5ug	Sigma	0.1	mg/ml	100	ng/ml
conA	Concanavalin A	TCR	C5275-5MG	Sigma	5	mg/ml	2500	ng/ml
DMF	Dimethyl fumarate	Nrf2	242926	Sigma	5	mg/ml	10000	ng/ml
EGCG	Epigallocatechin-3-gallate	anti-oxidative stress	sc-200802	Santa Cruz	10	mg/ml	45.84	ug/ml
FTY	Fingolimod	S1PR		Novartis	1	mg/ml	3000	ng/ml
H2O2	Hydrogen peroxide	oxidative stress	H3410	Sigma	330	mg/ml	17005	ng/ml
IFNB1a	Interferon beta 1a	Type I IFNR	101322	Merck	0.088	mg/ml	50	ng/ml
IFNG	Interferon gamma	Type II IFNR	I3265	Sigma	0.1	mg/ml	100	ng/ml
IL1A	Interleukin-1 alpha	IL1R pathway	200-01A	PeproTech	0.1	mg/ml	50	ng/ml
IL6	Interleukin-6	IL6R	200-06	PeproTech	0.1	mg/ml	100	ng/ml

INS	Insulin	InsulinR	I9278	Sigma	1.722	mg/ml	1722	ng/ml
LPS	Lipopolysaccharide	TLR4	L4391	Sigma	1	mg/ml	10000	ng/ml
NaCl	Sodium Chloride	PI3K	S5886	Sigma	11.7	mg/ml	2.34	mg/ml
PolyIC	Polyinosinic-Polycytidylic acid	TLR3	p0913-10mg	Sigma	10	mg/ml	10000	ng/ml
S1P	Sphingosine 1-phosphate	S1P	S9666	Sigma	0.125	mg/ml	100	ng/ml
Teriflu	Teriflunomide	pyrimidin synthesis<br>NFkB inhibitor	A77 1726	Calbiochem	10	mg/ml	13510	ng/ml
TNFA	Tumor necrosis factor alpha	TNF	300-01A	PeproTech	0.1	mg/ml	100	ng/ml
vitD3	vitamin D3	B-catenin	C9756	Sigma	1	mg/ml	500	ng/ml
BN201	Neuroprotective peptoid IGF-1 NA Bionure Farma	SGK	Under preparation	Bionure	1	mg/ml	5000	ng/ml

Table S4. References of the protein interactions of the Prior Knowledge Network (PKN). Table bibliography at the end of Additional file 2.

Nr.	Interaction	Reference
1	ABL1 $\longrightarrow$ ZAP70	[1]
2	AKT1 $\longrightarrow\!\!\!\!\dashv$ BAD	[2]
3	AKT1 $\longrightarrow$ CREB1	[3]
4	AKT1 $\longrightarrow\!\!\!\!\dashv$ FOXO1	[2]
5	AKT1 $\longrightarrow$ FOXO3	[4]
6	AKT1 $\longrightarrow$ GSK3A	[2], [5]
7	AKT1 $\longrightarrow$ GSK3B	[2], [5]
8	AKT1 $\longrightarrow$ IKKB	[6]
9	AKT1 $\longrightarrow\!\!\!\!\dashv$ P21	[2], [5]
10	AKT1 $\longrightarrow\!\!\!\!\dashv$ PSA6	[2], [5]
11	AKT1 $\longrightarrow\!\!\!\!\dashv$ RAF1	[7]
12	AKT1 $\longrightarrow\!\!\!\!\dashv$ SLP76	[8],<br>indirect interaction via ZAP70
13	AKT1 $\longrightarrow$ STAT1	[9]
14	AKT1 $\longrightarrow$ STAT3	[10]
15	AKT1 $\longrightarrow\!\!\!\!\dashv$ ZAP70	[8]
16	ANTICD3 $\longrightarrow$ TCR	Model Input
17	ANTICD3 $\longrightarrow$ WNK1	[11]
18	BAD $\longrightarrow\!\!\!\!\dashv$ BCLXL	[12], [13]
19	BCL10 $\longrightarrow$ CARD11A	[14], [15], [16]
20	BCLXL $\longrightarrow\!\!\!\!\dashv$ BID	[17]
21	BCLXL $\longrightarrow\!\!\!\!\dashv$ P53	[18]
22	BCR $\longrightarrow$ SYK	[19]
23	BDNF $\longrightarrow$ NTRK1	[20]
24	BID $\longrightarrow$ CYC1	[17]
25	BN201 $\longrightarrow$ SGK	[21]
26	CA $\longrightarrow$ CALM	[22]
27	CABIN $\longrightarrow\!\!\!\!\dashv$ CALCINEURIN	[23], [24], [25]
28	CALCINEURIN $\longrightarrow$ NFAT	[26], [27], [28]
29	CALM $\longrightarrow$ CALCINEURIN	[23], [24], [25]
30	CALM $\longrightarrow$ KCC2B	[29]
31	CALM $\longrightarrow$ KCC4	[30]
32	CARD11 $\longrightarrow$ CARD11A	[15], [14], [16]
33	CARD11A $\longrightarrow$ NEMO	[15], [14], [16]
34	CASP3 $\longrightarrow$ FAK1	[31]
35	CASP8 $\longrightarrow$ BID	[17]
36	CASP8 $\longrightarrow$ CASP3	[17]
37	CASP9 $\longrightarrow$ CASP3	[17]
38	CBL $\longrightarrow\!\!\!\!\dashv$ EGFR	[32], [33]

Nr.	Interaction	Reference
39	CBL ⟶ FYN	[34]
40	CBL ⟶ LCK	[35]
41	CBL ⊣ PGFRB	[36], [37]
42	CBL ⊣ PI3K	[38]
43	CBL ⊣ TCRP	[26], [39]
44	CBL ⊣ ZAP70	[1], [39]
45	CD19 ⟶ PI3K	[19]
46	CD28 ⟶ GADS	[40]
47	CD28 ⟶ GRB2	[41], [42]
48	CD28 ⟶ PI3K	[43], [44]
49	CD28 ⟶ X	[45]
50	CD4 ⊣ LCK	[46]
51	CD45 ⊣ FYN	[47]
52	CD45 ⊣ LCK	[46]
53	CD8 ⊣ LCK	[46]
54	CDC42 ⟶ MAP3K1	[48], [49]
55	CDC42 ⟶ SRE	[50]
56	CERAMIDE ⟶ PAR4	[51]
57	CONA ⟶ BCR	[52]
58	CONA ⟶ FCGR	[52]
59	CONA ⟶ INSR	[53]
60	CREB1 ⟶ CRE	[28]
61	CREB1 ⟶ JUN	[54], [55]
62	CSK ⟶ FYN	[46]
63	CSK ⟶ LCK	[46]
64	CYC1 ⟶ CASP9	[17]
65	DAG1 ⟶ GRP1	[56], [57]
66	DAG1 ⟶ PKC	[58]
67	DGK ⊣ DAG1	[59]
68	DMF ⟶ NF2L2	[60]
69	EGCG ⊣ EGFR	[61], [62], [63]
70	EGCG ⊣ INSR	[64]
71	EGCG ⊣ MK12	[61], [62], [63]
72	EGCG ⊣ MKO3	[61], [62], [63]
73	EGCG ⊣ PI3K	[61], [62], [63]
74	EGCG ⊣ RPS6KB1	[65]
75	EGCG ⊣ TF65	[61], [62]
76	EGF ⟶ EGFR	[32]
77	EGFR ⟶ CBL	[32, 66]
78	EGFR ⟶ GRB2	[67]

**Nr.**	**Interaction**	**Reference**
79	EGFR ⟶ INSR	[68]
80	FCGR ⟶ SHIP	[19]
81	FOS ⟶ JUN	[26], [28]
82	FYN ⊣ ABL1	[1]
83	FYN ⊣ FAK1	[69], [70]
84	FYN ⊣ PAG	[71]
85	FYN ⊣ TCRP	[72]
86	GAB2 ⊣ SLP76	[73, 74]
87	GADS ⟶ GAB2	[73]. [74]
88	GADS ⟶ SLP76	[75], [76]
89	GAP ⟶ RAS	[77]
90	GILENYA ⟶ S1PR1	[78]
91	GP130 ⟶ JAK1	[79], [80]
92	GP130 ⟶ PTN11	[81]
93	GRB2 ⟶ GAB2	[73], [74]
94	GRB2 ⟶ SOS	[82]
95	GRP1 ⟶ RAS	[71]
96	GSK3B ⊣ BCAT	[5]
97	GSK3B ⊣ CYC1	[5]
98	GSK3B ⊣ NFAT	[83]
99	H2O2 ⊣ KEAP1	[84]
100	H2O2 ⟶ P53	[85]
101	ICAM1 ⟶ PLCG1	[86]
102	IFNG ⟶ JAK1	[87]
103	IKBA ⟶ TF65	[26]
104	IKKB ⟶ IKBA	[26], [28]
105	IL1A ⟶ IL1R1	[88]
106	IL1R1 ⟶ MYD88	[88]
107	IL1R1 ⟶ TOLLIP	[88]
108	IL2 ⟶ JAK3	[89]
109	IL4 ⟶ JAK3	[89]
110	IL6 ⟶ GP130	[90]
111	INS ⟶ INSR	[91]
112	INSR ⟶ PI3K	[91]
113	INTEGRIN ⟶ ICAM1	[86]
114	IP3 ⟶ CA	[92]
115	IRAK4 ⟶ TRAF6	[88]
116	IRAKM ⊣ IL1R1	[88]
117	ITK ⟶ PLCG1	[93], [94]
118	JAK1 ⟶ AKT1	[10]

Nr.	Interaction	Reference
119	JAK1 $\longrightarrow$ PTN11	[81]
120	JAK1 $\longrightarrow$ STAT1	[80]
121	JAK1 $\longrightarrow$ STAT2	[95]
122	JAK1 $\longrightarrow$ STAT3	[10], [79], [80]
123	JAK1 $\longrightarrow$ STAT6	[89], [80]
124	JAK3 $\longrightarrow$ GAB2	[10]
125	JAK3 $\longrightarrow$ JAK1	[10]
126	JAK3 $\longrightarrow$ SYK	[89], [10]
127	JNK $\longrightarrow$ FOXO1	[96]
128	JNK $\longrightarrow$ FOXO3	[96]
129	JNK $\longrightarrow$ JUN	[28]
130	JUN $\longrightarrow$ CREB1	[55]
131	KCC2B $\longrightarrow$ IKKB	[15], [14], [16]
132	KCC4 $\longrightarrow\!\!\!\!\dashv$ CABIN	[97]
133	KEAP1 $\longrightarrow\!\!\!\!\dashv$ NF2L2	[84]
134	KPCZ $\longrightarrow$ GSK3B	[51]
135	KPCZ $\longrightarrow$ IKKB	[51]
136	LAT $\longrightarrow$ GAB2	[73], [74]
137	LAT $\longrightarrow$ GADS	[75], [76]
138	LAT $\longrightarrow$ GRB2	[98], [75]
139	LAT $\longrightarrow$ MAP4K1	[99]
140	LAT $\longrightarrow$ PLCB1	[100, 26]
141	LAT $\longrightarrow$ SH3BP2	[101]
142	LCK $\longrightarrow\!\!\!\!\dashv$ ABL1	[1]
143	LCK $\longrightarrow$ FYN	[102], [47]
144	LCK $\longrightarrow\!\!\!\!\dashv$ PI3K	[103]
145	LCK $\longrightarrow\!\!\!\!\dashv$ STAT3	[10]
146	LCK $\longrightarrow\!\!\!\!\dashv$ STAT5	[10]
147	LCK $\longrightarrow\!\!\!\!\dashv$ TCRP	[102], [47]
148	LCK $\longrightarrow\!\!\!\!\dashv$ TXK	[104]
149	LCK $\longrightarrow\!\!\!\!\dashv$ ZAP70	[46]
150	LPS $\longrightarrow$ IRAKM	[88]
151	LPS $\longrightarrow$ TLR4	[105]
152	MALT1 $\longrightarrow$ CARD11A	[15], [14], [16]
153	MAP2K3 $\longrightarrow$ MK12	[106]
154	MAP2K4 $\longrightarrow$ MAPK8	[107]
155	MAP2K4 $\longrightarrow$ MK12	[106]
156	MAP2K6 $\longrightarrow$ MK12	[108]
157	MAP3K1 $\longrightarrow$ MAP2K4	[109]
158	MAP3K1 $\longrightarrow$ MK12	[110]

Nr.	Interaction	Reference
159	MAP3K11 ⟶ MAP2K4	[111]
160	MAP4K1 ⟶ MAP3K1	[112]
161	MAP4K1 ⟶ MAP3K11	[111]
162	MAPK8 ⟶ JUN	[28]
163	MK12 ⟶ CREB1	[113]
164	MK12 ⟶ JNK	[114]
165	MK12 ⟶ RPS6KA5	[115]
166	MK12 ⟶ SGK	[116]
167	MK12 ⊣ TAK1	[88]
168	MKO3 ⟶ FOS	[26]
169	MKO3 ⟶ JUN	[117]
170	MKO3 ⟶ RPS6KA1	[118]
171	MKO3 ⊣ SHP1	[119]
172	MKO3 ⊣ ZAP70	[120] potential inhibition through specific phosphorylation site
173	MP2K1 ⟶ MKO3	[26], [28]
174	MYD88 ⟶ IRAK4	[88], [121]
175	NACL ⟶ MAP2K6	[122]
176	NEMO ⟶ IKKB	[15], [14], [16]
177	NF2L2 ⟶ CREB1	[123]
178	NF2L2 ⟶ JUN	[124]
179	NF2L2 ⟶ MRP2	[125]
180	NF2L2 ⟶ NQO1	[125]
181	NTRK2 ⟶ GRB2	[126]
182	P53 ⟶ BID	[18]
183	P53 ⟶ CASP8	[18]
184	P53 ⟶ CASP9	[18]
185	P53 ⟶ P21	[127]
186	P53 ⟶ PTEN	[18]
187	P53 ⟶ PUMA	[128]
188	PAG ⟶ CSK	[98], [75]
189	PAR4 ⊣ KPCZ	[51]
190	PDPK1 ⟶ AKT1	[129], [130], [30]
191	PDPK1 ⟶ PKC	[58]
192	PDPK1 ⟶ RPS6KB1	[131], [132]
193	PGFB ⟶ PGFRB	[133]
194	PGFRB ⟶ CBL	[36]
195	PGFRB ⟶ GRB2	[134]
196	PI3K ⟶ PIP3	[135], [136]

Nr.	Interaction	Reference
197	PI3K $\longrightarrow$ RPS6KB1	[137]
198	PIP3 $\longrightarrow$ AKT1	[138]
199	PIP3 $\longrightarrow$ ITK	[26], [76], [93]
200	PIP3 $\longrightarrow$ KPCZ	[10]
201	PIP3 $\longrightarrow$ PDPK1	[139]
202	PKC $\longrightarrow$ FAK1	[86]
203	PKC $\longrightarrow$ NEMO	[140]
204	PLCB1 $\longrightarrow$ PLCG1	[93], [94]
205	PLCG1 $\longrightarrow$ DAG1	[26]
206	PLCG1 $\longrightarrow$ IP3	[26], [76]
207	PLCG1 $\longrightarrow$ PKC	[86]
208	POLYIC $\longrightarrow$ TLR3	[141]
209	PTEN $\longrightarrow\!\!\!\!\dashv$ PIP3	[135], [136]
210	PTN11 $\longrightarrow$ GRB2	[142]
211	PUMA $\longrightarrow\!\!\!\!\dashv$ BCLXL	[128]
212	RAC1 $\longrightarrow$ MAP3K1	[48]
213	RAC1 $\longrightarrow$ MAP3K11	[143]
214	RAC1 $\longrightarrow$ SRE	[50]
215	RAF1 $\longrightarrow$ MP2K1	[144]
216	RAF1 $\longrightarrow$ SGK	[145]
217	RAS $\longrightarrow$ MAP3K1	[146]
218	RAS $\longrightarrow$ RAF1	[28]
219	REBIF $\longrightarrow$ JAK1	[147]
220	RPS6KA1 $\longrightarrow$ RS6	[118]
221	RPS6KA5 $\longrightarrow$ HSPB1	[148]
222	RPS6KB1 $\longrightarrow$ RS6	[118]
223	RS6 $\longrightarrow$ CREB1	[118]
224	S1P1 $\longrightarrow$ S1PR1	[149]
225	S1PR1 $\longrightarrow$ CERAMIDE	[150]
226	SGK $\longrightarrow$ CREB1	[151]
227	SGK $\longrightarrow\!\!\!\!\dashv$ FOXO1	[152]
228	SGK $\longrightarrow\!\!\!\!\dashv$ FOXO3	[4]
229	SGK $\longrightarrow$ GSK3B	[153]
230	SGK $\longrightarrow$ IKBA	[154]
231	SGK $\longrightarrow$ IKKB	[154]
232	SGK $\longrightarrow$ MKO3	[155]
233	SGK $\longrightarrow$ RPS6KB1	[156]
234	SH3BP2 $\longrightarrow$ VAV	[101]
235	SH3BP2 $\longrightarrow$ VAV3	[157]
236	SHC $\longrightarrow$ GRB2	[10]

Nr.	Interaction	Reference
237	SHIP ⊣ PIP3	[135], [136]
238	SHP1 ⟶ LCK	[46]
239	SHP1 ⊣ ZAP70	[158]
240	SLP76 ⟶ AKT1	[159]
241	SLP76 ⟶ ITK	[26], [76], [93]
242	SLP76 ⟶ VAV	[26]
243	SOCS1 ⊣ STAT1	[160]
244	SOCS3 ⊣ GP130	[161]
245	SOS ⟶ RAS	[56]
246	STAT1 ⟶ IRF1	[87]
247	STAT1 ⟶ PRDM1	[10]
248	STAT1 ⟶ SOCS1	[160]
249	STAT3 ⟶ BCLXL	[95]
250	STAT3 ⟶ FOS	[162]
251	STAT3 ⟶ PRDM1	[10]
252	STAT3 ⟶ SOCS3	[161]
253	STAT5 ⟶ BCLXL	[80]
254	STAT5 ⟶ PRDM1	[10]
255	SYK ⟶ PLCG1	[19]
256	SYK ⟶ SHC	[163]
257	SYK ⟶ STAT1	[164]
258	SYK ⟶ STAT3	[10]
259	SYK ⟶ STAT5	[10]
260	SYK ⟶ VAV	[19]
261	TAK1 ⟶ IKKB	[88]
262	TAK1 ⟶ MAP2K3	[88]
263	TAK1 ⟶ MAP2K4	[88]
264	TAK1 ⟶ MAP2K6	[88]
265	TCR ⊣ FYN	[72]
266	TCR ⊣ PAG	[71]
267	TCR ⟶ TCRP	[47]
268	TCRP ⟶ DGK	[165]
269	TCRP ⟶ ZAP70	[1]
270	TERIFLUNOMIDE ⊣ TF65	[166]
271	TF65 ⟶ IKBA	[88]
272	TLR3 ⟶ TRAF6	[121]
273	TLR4 ⟶ MYD88	[121]
274	TNFA ⟶ TRADD	[167], [168]
275	TOLLIP ⊣ IL1R1	[88]
276	TRADD ⟶ CASP8	[167], [168]

Nr.	Interaction	Reference
277	TRADD ⟶ TRAF2	[169]
278	TRAF2 ⟶ IKKB	[169], [168]
279	TRAF2 ⟶ MAP3K1	[168]
280	TRAF6 ⟶ TAK1	[88]
281	TXK ⟶ PLCG1	[93], [94]
282	VAV ⟶ PKC	[170]
283	VAV ⟶ PLCG1	[93], [94]
284	VAV ⟶ RAC1	[157]
285	VAV3 ⟶ RAC1	[157]
286	VDR ⟶ STAT1	[171]
287	VITD3 ⟶ VDR	[171]
288	X ⟶ PI3K	[45]
289	X ⟶ VAV	[45]
290	ZAP70 ⟶ GAB2	[76], [73], [74]
291	ZAP70 ⟶ LAT	[26]
292	ZAP70 ⟶ MK12	[172]
293	ZAP70 ⟶ SH3BP2	[101]
294	ZAP70 ⟶ SLP76	[75], [76]

Table S5. List of the phosphoproteins selected from the literature search, the stimuli applied and the MS drugs used for building the Prior Knowledge Network (PKN)

No	Model Name	Name	UniProt-ID / ChEMBL-ID
1	ABL1	Tyrosine-protein kinase ABL1	P00519
2	AKT1	RAC-alpha serine/threonine-protein kinase	P31749
3	ANTICD3	Antibody against human CD3	
4	BAD	Bcl2-associated agonist of cell death	Q92934
5	BCAT	Catenin beta-1	P35222
6	BCL10	B-cell lymphoma/leukemia 10	O95999
7	BCLXL	Bcl-2-like protein 1	Q07817
8	BCR	B-cell receptor	
9	BDNF	Brain-derived neurotrophic factor	P23560
10	BID	BH3-interacting domain death agonist	P55957
11	BN201	Drug candidate by Bionure	
12	CA	Calcium	CHEMBL2146121
13	CABIN	Calcineurin-binding protein cabin-1	Q9Y6J0
14	CALCINEURIN	Calcineurin phosphatase family	
15	CALM	Calmodulin	P62158
16	CARD11	Caspase recruitment domain-containing protein 11	Q9BXL7
17	CARD11A	Complex of BCL10, CARD11, and MALT1	
18	CASP3	Caspase-3	P42574
19	CASP8	Caspase-8	Q14790
20	CASP9	Caspase-9	P55211
21	CBL	E3 ubiquitin-protein ligase CBL	P22681
22	CD19	B-lymphocyte antigen CD19	P15391
23	CD28	T-cell-specific surface glycoprotein CD28	P10747
24	CD4	T-cell surface glycoprotein CD4	P01730
25	CD45	Receptor-type tyrosine-protein phosphatase C	P08575
26	CD8	T-cell surface glycoprotein CD8 alpha / beta chain	P01732 / P10966
27	CDC42	Cell division control protein 42 homolog	P60953
28	CERAMIDE	Lipid molecules involved in lipid signalling	
29	CONA	Concavallin A	CHEMBL3215495
30	CRE	cAMP responsive element	
31	CREB1	Cyclic AMP-responsive element-binding protein 1	P16220
32	CSK	Tyrosine-protein kinase CSK	P41240
33	CYC1	Cytochrome c1, heme protein, mitochondrial	P08574
34	DAG1	Diacylglycerol	
35	DGK	Diacylglycerol kinase family	
36	DMF	Dimethyl Fumarate	CHEMBL2107333
37	EGCG	(-)-Epigallocatechin Gallate	CHEMBL297453
38	EGF	Pro-epidermal growth factor	P01133
39	EGFR	Epidermal growth factor receptor	P00533
40	FAK1	Focal adhesion kinase 1	Q05397
41	FCGR	Immunoglobulin gamma Fc receptor family	
42	FOS	Proto-oncogene c-Fos	P01100
43	FOXO1	Forkhead box protein O1	Q12778
44	FOXO3	Forkhead box protein O3	O43524
45	FYN	Tyrosine-protein kinase Fyn	P06241
46	GAB2	GRB2-associated-binding protein 2	Q9UQC2
47	GADS	GRB2-related adapter protein 2	O75791
48	GAP	Ras GTPase-activating protein 1	P20936
49	GILENYA	Gilenya, Fingolimod	CHEMBL314854
50	GP130	Interleukin-6 receptor subunit beta	P40189
51	GRB2	Growth factor receptor-bound protein 2	P62993
52	GRP1	RAS guanyl-releasing protein 1	O95267
53	GSK3A	Glycogen synthase kinase-3 alpha	P49840
54	GSK3B	Glycogen synthase kinase-3 beta	P49841
55	H2O2	Hydrogen Peroxide	CHEMBL71595
56	HSPB1	Heat shock protein beta-1	P04792
57	ICAM1	Intercellular adhesion molecule 1	P05362
58	IFNG	Interferon gamma	P01579
59	IKBA	NF-kappa-B inhibitor alpha	P25963
60	IKKB	Inhibitor of nuclear factor kappa-B kinase subunit beta	O14920
61	IL1A	Interleukin-1 alpha	P01583
62	IL1R1	Interleukin-1 receptor type 1	P14778
63	IL2	Interleukin-2	P60568
64	IL4	Interleukin-4	P05112
65	IL6	Interleukin-6	P05231
66	INS	Insulin	P01308
67	INSR	Insulin receptor	P06213
68	INTEGRIN	Integrin protein family	
69	IP3	Ionositol 1,4,5-Triphosphate	CHEMBL279107
70	IRAK4	Interleukin-1 receptor-associated kinase 4	Q9NWZ3
71	IRAKM	Interleukin-1 receptor-associated kinase 3	Q9Y616
72	IRF1	Interferon regulatory factor 1	P10914
73	ITK	Tyrosine-protein kinase ITK/TSK	Q08881
74	JAK1	Tyrosine-protein kinase JAK1	P23458

75	JAK3	Tyrosine-protein kinase JAK3	P52333
76	JNK	Mitogen-activated protein kinase 8 / 9 / 10	P45983/P45984/P53779
77	JUN	Transcription factor AP-1	P05412
78	KCC2B	Calcium/calmodulin-dependent protein kinase type II subunit beta	Q13554
79	KCC4	Calcium/calmodulin-dependent protein kinase type IV	Q16566
80	KEAP1	Kelch-like ECH-associated protein 1	Q14145
81	KPCZ	Protein kinase C zeta type	Q05513
82	LAT	Linker for activation of T-cells family member 1	O43561
83	LCK	Tyrosine-protein kinase Lck	P06239
84	LPS	Lipopolysaccharides	
85	MALT1	Mucosa-associated lymphoid tissue lymphoma translocation protein 1	Q9UDY8
86	MAP2K3	Dual specificity mitogen-activated protein kinase kinase 3	P46734
87	MAP2K4	Dual specificity mitogen-activated protein kinase kinase 4	P45985
88	MAP2K6	Dual specificity mitogen-activated protein kinase kinase 6	P52564
89	MAP3K1	Mitogen-activated protein kinase kinase kinase 1	Q13233
90	MAP3K11	Mitogen-activated protein kinase kinase kinase 11	Q16584
91	MAP4K1	Mitogen-activated protein kinase kinase kinase kinase 1	Q92918
92	MAPK8	Mitogen-activated protein kinase 8	P45983
93	MK12	Mitogen-activated protein kinase 12	P53778
94	MK03	Mitogen-activated protein kinase 3	P27361
95	MP2K1	Dual specificity mitogen-activated protein kinase kinase 1	Q02750
96	MRP2	Canalicular multispecific organic anion transporter 1	Q92887
97	MYD88	Myeloid differentiation primary response protein MyD88	Q99836
98	NACL	Sodium chloride	CHEMBL1200574
99	NEMO	NF-kappa-B essential modulator	Q9Y6K9
100	NF2L2	Nuclear factor erythroid 2-related factor 2	Q16236
101	NFAT	Nuclear factor of activated T-cells, C1 / C2 / C3 / C4	O95644/Q13469/Q12968/Q14934
102	NQO1	NAD(P)H dehydrogenase [quinone] 1	P15559
103	NTRK1	High affinity nerve growth factor receptor	P04629
104	P21	Cyclin-dependent kinase inhibitor 1	P38936
105	P53	Cellular tumor antigen p53	P04637
106	PAG	Phosphoprotein associated with glycosphingolipid-enriched microdomains 1	Q9NWQ8
107	PAR4	PRKC apoptosis WT1 regulator protein	Q96IZ0
108	PDPK1	3-phosphoinositide-dependent protein kinase 1	O15530
109	PGFB	Platelet-derived growth factor subunit B	P01127
110	PGFRB	Platelet-derived growth factor receptor beta	P09619
111	PI3K	Phosphoinositide 3-kinase family	
112	PIP3	Phosphatidylinositol (3,4,5) - trisphosphate	CHEMBL1685065
113	PKC	Protein kinase C family	
114	PLCB1	1-phosphatidylinositol 4,5-bisphosphate phosphodiesterase beta-1	Q9NQ66
115	PLCG1	1-phosphatidylinositol 4,5-bisphosphate phosphodiesterase gamma-1	P19174
116	POLYIC	Polyinosinic:polycytidylic acid	
117	PRDM1	PR domain zinc finger protein 1	O75626
118	PSA6	Cyclin-dependent kinase inhibitor 1B	P46527
119	PTEN	Phosphatidylinositol 3,4,5-trisphosphate 3-phosphatase and dual-specificity protein phosphatase PTEN	P60484
120	PTN11	Tyrosine-protein phosphatase non - receptor type 11	Q06124
121	PUMA	Bcl-2-binding component 3	Q9BXH1/Q96PG8
122	RAC1	Ras-related C3 botulinum toxin substrate 1	P63000
123	RAF1	RAF proto-oncogene serine/threonine-protein kinase	P04049
124	RAS	GTPase Kras / Hras / NRas	P01116/P01112/P01111
125	REBIF	Interferon beta-1a	CHEMBL1201562
126	RPS6KA1	Ribosomal protein S6 kinase alpha-1	Q15418
127	RPS6KA5	Ribosomal protein S6 kinase alpha-5	O75582
128	RPS6KB1	Ribosomal protein S6 kinase beta-1	P23443
129	RS6	40S ribosomal protein S6	P62753
130	S1P1	Sphingosine-1-phosphate	CHEMBL78494
131	S1PR1	Sphingosine 1-phosphate receptor 1	P21453
132	SGK	Serine/threonine-protein kinase Sgk1/2/3	O00141/Q9HBY8/Q96BR1
133	SH3BP2	SH3 domain-binding protein 2	P78314
134	SHC	SHC-transforming protein 1	P29353
135	SHIP	Phosphatidylinositol 3,4,5-trisphosphate 5-phosphatase 1	Q92835
136	SHP1	Protein-tyrosine phosphatase	P29350
137	SLP76	Lymphocyte cytosolic protein 2	Q13094
138	SOCS1	Suppressor of cytokine signaling 1	O15524
139	SOCS3	Suppressor of cytokine signaling 3	O14543
140	SOS	Son of sevenless homolog 1	Q07889
141	SRE	Serum response element	
142	STAT1	Signal transducer and activator of transcription 1	P42224
143	STAT2	Signal transducer and activator of transcription 2	P52630
144	STAT3	Signal transducer and activator of transcription 3	P40763
145	STAT5	Signal transducer and activator of transcription 5A / 5B	P42229/P51692
146	STAT6	Signal transducer and activator of transcription 6	P42226
147	SYK	Tyrosine-protein kinase SYK	P43405
148	TAK1	Mitogen-activated protein kinase kinase kinase 7	O43318
149	TCR	T-cell Receptor	

150	TCRP	T-cell Receptor phosphorylated	
151	TERIFLUNOMIDE	Teriflunomide, Aubagio	CHEMBL973
152	TF65	Transcription factor p65	Q04206
153	TLR3	Toll-like receptor 3	O15455
154	TLR4	Toll-like receptor 4	O00206
155	TNFA	Tumor necrosis factor	P01375
156	TOLLIP	Toll-interacting protein	Q9H0E2
157	TRADD	Tumor necrosis factor receptor type 1-associated DEATH domain protein	Q15628
158	TRAF2	TNF receptor-associated factor 2	Q12933
159	TRAF6	TNF receptor-associated factor 6	Q9Y4K3
160	TXK	Tyrosine-protein kinase TXK	P42681
161	VAV	Proto-oncogene vav	P15498
162	VAV3	Guanine nucleotide exchange factor VAV3	Q9UKW4
163	VDR	Vitamin D3 receptor	P11473
164	VITD3	Vitamin D 3, Cholecalciferol	CHEMBL1042
165	WNK1	Serine/threonine-protein kinase WNK1	Q9H4A3
166	X	Non-identified kinase involved in CD28- mediated signalling, see Saez-Rodriguez2007	
167	ZAP70	Tyrosine-protein kinase ZAP-70	P43403

Table S6. List of co-druggable interactions and their co-druggability score

No	ECGC		FTY		GA		IFNbeta		NTZ	
1	!LCK=PI3K	-0.41	!STAT3=GP130	-0.52	CONA=GRB2	-0.36	!CBL=ZAP70	-0.19	!ANTICD3=PAG	-0.06
2	!LCK=STAT3	-0.33	AKT1=GSK3A	-0.19	DAG1=RAS	-0.23	BDNF=GRB2	0.00	ANTICD3=WNK1	0.00
3	AKT1=GSK3A	-0.34	ANTICD3=TCRP	-0.17	IL1A=IL1R1	-0.14	CONA=VAV	0.00	BDNF=GRB2	-0.05
4	BDNF=GRB2	-0.23	BDNF=GRB2	-0.14	IL1R1=TAK1	-0.16	GP130=JAK1	-0.11	CONA=STAT1	-0.06
5	CONA=GRB2	-0.33	IL1A=IL1R1	-0.11	MAP2K4=MK12	-0.16	JAK1=STAT1	-0.06	GP130=JAK1	-0.10
6	CONA=VAV	-0.42	IL1R1=TAK1	-0.12	MAP3K1=MAP2K4	-0.19	LPS=TAK1	-0.14	INS=PI3K	0.00
7	GRB2=RAS	-0.28	IL6=GP130	-0.43	NACL=MK12	-0.12	MAP3K1=MAP2K4	-0.08	MAP2K4=MK12	-0.15
8	JAK1=STAT3	0.00	MK12=HSPB1	-0.28	PAG=LCK	-0.22	MAP3K1=MK12	-0.12	MAP3K1=MAP2K4	-0.08
9	PI3K=PIP3	-0.36	NACL=MK12	0.00	PIP3=PLCG1	-0.26	NACL=MK12	-0.16	NACL=MK12	-0.06
10	PIP3=AKT1	-0.31	RAC1=MAP3K1	-0.19	PLCG1=DAG1	-0.26	PAG=LCK	-0.13	REBIF=JAK1	-0.05
11	RAS=MAP3K1	-0.57	REBIF=JAK1	-0.09	RAF1=SGK	-0.26	SLP76=AKT1	-0.14	SLP76=AKT1	-0.10
12	TAK1=MAP2K4	-0.36	TCRP=ZAP70	-0.28	SLP76=AKT1	-0.28	ZAP70=GRB2	0.00	TAK1=MK12	-0.15
13	VAV=RAC1	-0.37			ZAP70=SLP76	-0.26	ZAP70=SLP76	0.00		

Table S7. Predicted targets for combination therapy

Each row is a co-druggable reaction i.e. with a negative drug score (Figure 1 and Table 1) for which a path could identified between the denoted stimuli and readout. Reaction: Source=Target indicates activation, ! indicates inhibition. Boolean network activity represents the active (1) and inactive (0) reactions for each subgroup (Additional file 3: Figure S3). Group network activity denotes the mean signaling activity of this reaction over all donors within each drug-treated subgroup (Additional file 3: Figure S2). For a combination therapy, each stimulus indicated in a given row could be combined with the treatment indicated as a header of each subgroup.

	Reaction	Stimulus	Readout	drugScore	Boolean network activity	Group network activity
**EGCG**						
1	AKT1=GSK3A	INS	GSK3A	-0.34	0	0.00
2	BDNF=GRB2	BDNF	MK12	-0.23	1	1.00
3	BDNF=GRB2	BDNF	MP2K1	-0.23	1	1.00
4	BDNF=GRB2	BDNF	MKO3	-0.23	1	1.00
5	CONA=GRB2	CONA	MK12	-0.33	1	0.67
6	CONA=GRB2	CONA	MP2K1	-0.33	1	0.67
7	CONA=GRB2	CONA	MKO3	-0.33	1	0.67
8	GRB2=RAS	BDNF	MK12	-0.28	1	1.00
9	GRB2=RAS	BDNF	MP2K1	-0.28	1	1.00
10	GRB2=RAS	BDNF	MKO3	-0.28	1	1.00
11	GRB2=RAS	IL1A	MK12	-0.28	1	1.00
12	GRB2=RAS	IL1A	MP2K1	-0.28	1	1.00
13	GRB2=RAS	IL1A	MKO3	-0.28	1	1.00
14	GRB2=RAS	LPS	MK12	-0.28	1	1.00
15	GRB2=RAS	LPS	MP2K1	-0.28	1	1.00
16	GRB2=RAS	LPS	MKO3	-0.28	1	1.00
17	GRB2=RAS	POLYIC	MK12	-0.28	1	1.00
18	GRB2=RAS	POLYIC	MP2K1	-0.28	1	1.00
19	GRB2=RAS	POLYIC	MKO3	-0.28	1	1.00
20	GRB2=RAS	CONA	MK12	-0.28	1	1.00
21	GRB2=RAS	CONA	MP2K1	-0.28	1	1.00
22	GRB2=RAS	CONA	MKO3	-0.28	1	1.00
23	GRB2=RAS	TNFA	MK12	-0.28	1	1.00
24	GRB2=RAS	TNFA	MP2K1	-0.28	1	1.00
25	GRB2=RAS	TNFA	MKO3	-0.28	1	1.00
26	JAK1=STAT3	REBIF	STAT3	0.00	1	0.67
27	JAK1=STAT3	IL6	STAT3	0.00	1	0.67
28	PI3K=PIP3	INS	AKT1	-0.36	0	0.33
29	PI3K=PIP3	INS	GSK3A	-0.36	0	0.33
30	PIP3=AKT1	INS	AKT1	-0.31	0	0.00
31	PIP3=AKT1	INS	GSK3A	-0.31	0	0.00
32	RAS=MAP3K1	BDNF	MK12	-0.57	1	1.00
33	RAS=MAP3K1	BDNF	MP2K1	-0.57	1	1.00
34	RAS=MAP3K1	BDNF	MKO3	-0.57	1	1.00
35	RAS=MAP3K1	IL1A	MK12	-0.57	1	1.00
36	RAS=MAP3K1	IL1A	MP2K1	-0.57	1	1.00

36	RAS=MAP3K1	IL1A	MP2K1	-0.57	1	1.00
37	RAS=MAP3K1	IL1A	MKO3	-0.57	1	1.00
38	RAS=MAP3K1	LPS	MK12	-0.57	1	1.00
39	RAS=MAP3K1	LPS	MP2K1	-0.57	1	1.00
40	RAS=MAP3K1	LPS	MKO3	-0.57	1	1.00
41	RAS=MAP3K1	POLYIC	MK12	-0.57	1	1.00
42	RAS=MAP3K1	POLYIC	MP2K1	-0.57	1	1.00
43	RAS=MAP3K1	POLYIC	MKO3	-0.57	1	1.00
44	RAS=MAP3K1	CONA	MK12	-0.57	1	1.00
45	RAS=MAP3K1	CONA	MP2K1	-0.57	1	1.00
46	RAS=MAP3K1	CONA	MKO3	-0.57	1	1.00
47	RAS=MAP3K1	TNFA	MK12	-0.57	1	1.00
48	RAS=MAP3K1	TNFA	MP2K1	-0.57	1	1.00
49	RAS=MAP3K1	TNFA	MKO3	-0.57	1	1.00
50	TAK1=MAP2K4	IL1A	MK12	-0.36	1	0.67
51	TAK1=MAP2K4	IL1A	MP2K1	-0.36	1	0.67
52	TAK1=MAP2K4	IL1A	MKO3	-0.36	1	0.67
53	TAK1=MAP2K4	LPS	MK12	-0.36	1	0.67
54	TAK1=MAP2K4	LPS	MP2K1	-0.36	1	0.67
55	TAK1=MAP2K4	LPS	MKO3	-0.36	1	0.67
56	TAK1=MAP2K4	POLYIC	MK12	-0.36	1	0.67
57	TAK1=MAP2K4	POLYIC	MP2K1	-0.36	1	0.67
58	TAK1=MAP2K4	POLYIC	MKO3	-0.36	1	0.67
**FTY**						
1	AKT1=GSK3A	BDNF	GSK3A	-0.19	0	0.15
2	AKT1=GSK3A	NACL	GSK3A	-0.19	0	0.15
3	AKT1=GSK3A	IL1A	GSK3A	-0.19	0	0.15
4	AKT1=GSK3A	INS	GSK3A	-0.19	0	0.15
5	AKT1=GSK3A	LPS	GSK3A	-0.19	0	0.15
6	AKT1=GSK3A	POLYIC	GSK3A	-0.19	0	0.15
7	AKT1=GSK3A	CONA	GSK3A	-0.19	0	0.15
8	AKT1=GSK3A	TNFA	GSK3A	-0.19	0	0.15
9	BDNF=GRB2	BDNF	HSPB1	-0.14	0	0.38
10	BDNF=GRB2	BDNF	AKT1	-0.14	0	0.38
11	BDNF=GRB2	BDNF	MK12	-0.14	0	0.38
12	BDNF=GRB2	BDNF	GSK3A	-0.14	0	0.38
13	BDNF=GRB2	BDNF	MP2K1	-0.14	0	0.38
14	BDNF=GRB2	BDNF	MKO3	-0.14	0	0.38
15	BDNF=GRB2	BDNF	STAT5	-0.14	0	0.38
16	IL1A=IL1R1	IL1A	HSPB1	-0.11	1	0.54
17	IL1A=IL1R1	IL1A	AKT1	-0.11	1	0.54
18	IL1A=IL1R1	IL1A	MK12	-0.11	1	0.54
19	IL1A=IL1R1	IL1A	GSK3A	-0.11	1	0.54
20	IL1A=IL1R1	IL1A	MP2K1	-0.11	1	0.54
21	IL1A=IL1R1	IL1A	MKO3	-0.11	1	0.54
22	IL1A=IL1R1	IL1A	STAT5	-0.11	1	0.54
23	IL1R1=TAK1	IL1A	HSPB1	-0.12	1	0.54
24	IL1R1=TAK1	IL1A	AKT1	-0.12	1	0.54
25	IL1R1=TAK1	IL1A	MK12	-0.12	1	0.54
26	IL1R1=TAK1	IL1A	GSK3A	-0.12	1	0.54
27	IL1R1=TAK1	IL1A	MP2K1	-0.12	1	0.54
28	IL1R1=TAK1	IL1A	MKO3	-0.12	1	0.54
29	IL1R1=TAK1	IL1A	STAT5	-0.12	1	0.54

30	IL6=GP130	IL6	STAT6	-0.43	0	0.31
31	MK12=HSPB1	BDNF	HSPB1	-0.28	1	0.85
32	MK12=HSPB1	NACL	HSPB1	-0.28	1	0.85
33	MK12=HSPB1	IL1A	HSPB1	-0.28	1	0.85
34	MK12=HSPB1	INS	HSPB1	-0.28	1	0.85
35	MK12=HSPB1	LPS	HSPB1	-0.28	1	0.85
36	MK12=HSPB1	POLYIC	HSPB1	-0.28	1	0.85
37	MK12=HSPB1	CONA	HSPB1	-0.28	1	0.85
38	MK12=HSPB1	TNFA	HSPB1	-0.28	1	0.85
39	NACL=MK12	NACL	HSPB1	0.00	0	0.31
40	NACL=MK12	NACL	AKT1	0.00	0	0.31
41	NACL=MK12	NACL	MK12	0.00	0	0.31
42	NACL=MK12	NACL	GSK3A	0.00	0	0.31
43	NACL=MK12	NACL	MP2K1	0.00	0	0.31
44	NACL=MK12	NACL	MKO3	0.00	0	0.31
45	NACL=MK12	NACL	STAT5	0.00	0	0.31
46	RAC1=MAP3K1	CONA	HSPB1	-0.19	1	0.46
47	RAC1=MAP3K1	CONA	AKT1	-0.19	1	0.46
48	RAC1=MAP3K1	CONA	MK12	-0.19	1	0.46
49	RAC1=MAP3K1	CONA	GSK3A	-0.19	1	0.46
50	RAC1=MAP3K1	CONA	MP2K1	-0.19	1	0.46
51	RAC1=MAP3K1	CONA	MKO3	-0.19	1	0.46
52	RAC1=MAP3K1	CONA	STAT5	-0.19	1	0.46
53	REBIF=JAK1	REBIF	STAT6	-0.09	1	0.54
**GA**						
1	CONA=GRB2	CONA	MP2K1	-0.36	1	0.70
2	CONA=GRB2	CONA	MKO3	-0.36	1	0.70
3	DAG1=RAS	INS	MP2K1	-0.23	0	0.30
4	DAG1=RAS	INS	MKO3	-0.23	0	0.30
5	IL1A=IL1R1	IL1A	MK12	-0.14	1	0.50
6	IL1A=IL1R1	IL1A	MKO3	-0.14	1	0.50
7	IL1R1=TAK1	IL1A	MK12	-0.16	1	0.50
8	IL1R1=TAK1	IL1A	MKO3	-0.16	1	0.50
9	MAP2K4=MK12	TNFA	MK12	-0.16	0	0.20
10	MAP2K4=MK12	TNFA	MKO3	-0.16	0	0.20
11	MAP3K1=MAP2K4	TNFA	MK12	-0.19	0	0.10
12	MAP3K1=MAP2K4	TNFA	MKO3	-0.19	0	0.10
13	NACL=MK12	NACL	MK12	-0.12	0	0.20
14	NACL=MK12	NACL	MKO3	-0.12	0	0.20
15	PIP3=PLCG1	INS	MP2K1	-0.26	0	0.10
16	PIP3=PLCG1	INS	MKO3	-0.26	0	0.10
17	PLCG1=DAG1	INS	MP2K1	-0.26	0	0.30
18	PLCG1=DAG1	INS	MKO3	-0.26	0	0.30
19	RAF1=SGK	ANTICD3	MKO3	-0.26	1	0.50
20	RAF1=SGK	BDNF	MKO3	-0.26	1	0.50
21	RAF1=SGK	INS	MKO3	-0.26	1	0.50
22	RAF1=SGK	CONA	MKO3	-0.26	1	0.50
23	SLP76=AKT1	ANTICD3	AKT1	-0.28	1	0.60
24	SLP76=AKT1	ANTICD3	STAT1	-0.28	1	0.60
25	SLP76=AKT1	ANTICD3	GSK3A	-0.28	1	0.60
26	ZAP70=SLP76	ANTICD3	AKT1	-0.26	1	0.60
27	ZAP70=SLP76	ANTICD3	STAT1	-0.26	1	0.60
28	ZAP70=SLP76	ANTICD3	GSK3A	-0.26	1	0.60

IFNb						
1	!CBL=ZAP70	EGCG	HSPB1	-0.19	1	0.50
2	!CBL=ZAP70	EGCG	AKT1	-0.19	1	0.50
3	!CBL=ZAP70	EGCG	MK12	-0.19	1	0.50
4	!CBL=ZAP70	EGCG	GSK3A	-0.19	1	0.50
5	!CBL=ZAP70	EGCG	MP2K1	-0.19	1	0.50
6	!CBL=ZAP70	EGCG	MKO3	-0.19	1	0.50
7	BDNF=GRB2	BDNF	HSPB1	0.00	1	0.50
8	BDNF=GRB2	BDNF	MK12	0.00	1	0.50
9	BDNF=GRB2	BDNF	MP2K1	0.00	1	0.50
10	BDNF=GRB2	BDNF	MKO3	0.00	1	0.50
11	GP130=JAK1	IL6	STAT1	-0.11	1	0.96
12	GP130=JAK1	IL6	STAT3	-0.11	1	0.96
13	JAK1=STAT1	REBIF	STAT1	-0.06	1	0.67
14	JAK1=STAT1	IFNG	STAT1	-0.06	1	0.67
15	JAK1=STAT1	IL6	STAT1	-0.06	1	0.67
16	LPS=TAK1	LPS	HSPB1	-0.14	1	0.96
17	LPS=TAK1	LPS	MK12	-0.14	1	0.96
18	LPS=TAK1	LPS	MKO3	-0.14	1	0.96
19	MAP3K1=MAP2K4	ANTICD3	HSPB1	-0.08	0	0.21
20	MAP3K1=MAP2K4	ANTICD3	MK12	-0.08	0	0.21
21	MAP3K1=MAP2K4	ANTICD3	MKO3	-0.08	0	0.21
22	MAP3K1=MAP2K4	BDNF	HSPB1	-0.08	0	0.21
23	MAP3K1=MAP2K4	BDNF	MK12	-0.08	0	0.21
24	MAP3K1=MAP2K4	BDNF	MKO3	-0.08	0	0.21
25	MAP3K1=MAP2K4	INS	HSPB1	-0.08	0	0.21
26	MAP3K1=MAP2K4	INS	MK12	-0.08	0	0.21
27	MAP3K1=MAP2K4	INS	MKO3	-0.08	0	0.21
28	MAP3K1=MAP2K4	EGCG	HSPB1	-0.08	0	0.21
29	MAP3K1=MAP2K4	EGCG	MK12	-0.08	0	0.21
30	MAP3K1=MAP2K4	EGCG	MKO3	-0.08	0	0.21
31	MAP3K1=MAP2K4	CONA	HSPB1	-0.08	0	0.21
32	MAP3K1=MAP2K4	CONA	MK12	-0.08	0	0.21
33	MAP3K1=MAP2K4	CONA	MKO3	-0.08	0	0.21
34	MAP3K1=MAP2K4	TNFA	HSPB1	-0.08	0	0.21
35	MAP3K1=MAP2K4	TNFA	MK12	-0.08	0	0.21
36	MAP3K1=MAP2K4	TNFA	MKO3	-0.08	0	0.21
37	MAP3K1=MK12	ANTICD3	HSPB1	-0.12	1	0.79
38	MAP3K1=MK12	ANTICD3	MK12	-0.12	1	0.79
39	MAP3K1=MK12	ANTICD3	MKO3	-0.12	1	0.79
40	MAP3K1=MK12	BDNF	HSPB1	-0.12	1	0.79
41	MAP3K1=MK12	BDNF	MK12	-0.12	1	0.79
42	MAP3K1=MK12	BDNF	MKO3	-0.12	1	0.79
43	MAP3K1=MK12	INS	HSPB1	-0.12	1	0.79
44	MAP3K1=MK12	INS	MK12	-0.12	1	0.79
45	MAP3K1=MK12	INS	MKO3	-0.12	1	0.79
46	MAP3K1=MK12	EGCG	HSPB1	-0.12	1	0.79
47	MAP3K1=MK12	EGCG	MK12	-0.12	1	0.79
48	MAP3K1=MK12	EGCG	MKO3	-0.12	1	0.79
49	MAP3K1=MK12	CONA	HSPB1	-0.12	1	0.79
50	MAP3K1=MK12	CONA	MK12	-0.12	1	0.79
51	MAP3K1=MK12	CONA	MKO3	-0.12	1	0.79
52	MAP3K1=MK12	TNFA	HSPB1	-0.12	1	0.79

53	MAP3K1=MK12	TNFA	MK12	-0.12	1	0.79
54	MAP3K1=MK12	TNFA	MKO3	-0.12	1	0.79
55	NACL=MK12	NACL	HSPB1	-0.16	0	0.17
56	NACL=MK12	NACL	MK12	-0.16	0	0.17
57	NACL=MK12	NACL	MKO3	-0.16	0	0.17
58	SLP76=AKT1	ANTICD3	AKT1	-0.14	1	0.46
59	SLP76=AKT1	ANTICD3	GSK3A	-0.14	1	0.46
60	SLP76=AKT1	EGCG	AKT1	-0.14	1	0.46
61	SLP76=AKT1	EGCG	GSK3A	-0.14	1	0.46
62	ZAP70=GRB2	ANTICD3	HSPB1	0.00	0	0.33
63	ZAP70=GRB2	ANTICD3	MK12	0.00	0	0.33
64	ZAP70=GRB2	ANTICD3	MP2K1	0.00	0	0.33
65	ZAP70=GRB2	ANTICD3	MKO3	0.00	0	0.33
66	ZAP70=GRB2	EGCG	HSPB1	0.00	0	0.33
67	ZAP70=GRB2	EGCG	MK12	0.00	0	0.33
68	ZAP70=GRB2	EGCG	MP2K1	0.00	0	0.33
69	ZAP70=GRB2	EGCG	MKO3	0.00	0	0.33
70	ZAP70=SLP76	ANTICD3	AKT1	0.00	1	0.38
71	ZAP70=SLP76	ANTICD3	GSK3A	0.00	1	0.38
72	ZAP70=SLP76	EGCG	AKT1	0.00	1	0.38
73	ZAP70=SLP76	EGCG	GSK3A	0.00	1	0.38
**NTZ**						
1	!ANTICD3=PAG	ANTICD3	STAT5	-0.06	1	0.42
2	ANTICD3=WNK1	ANTICD3	WNK1	0.00	1	0.42
3	BDNF=GRB2	BDNF	MP2K1	-0.05	1	0.47
4	CONA=STAT1	CONA	STAT1	-0.06	1	0.42
5	GP130=JAK1	IL6	STAT1	-0.10	1	0.95
6	MAP2K4=MK12	TNFA	HSPB1	-0.15	0	0.21
7	MAP2K4=MK12	TNFA	MK12	-0.15	0	0.21
8	MAP2K4=MK12	TNFA	MKO3	-0.15	0	0.21
9	MAP2K4=MK12	TNFA	STAT5	-0.15	0	0.21
10	MAP3K1=MAP2K4	TNFA	HSPB1	-0.08	0	0.21
11	MAP3K1=MAP2K4	TNFA	MK12	-0.08	0	0.21
12	MAP3K1=MAP2K4	TNFA	MKO3	-0.08	0	0.21
13	MAP3K1=MAP2K4	TNFA	STAT5	-0.08	0	0.21
14	NACL=MK12	NACL	HSPB1	-0.06	0	0.26
15	NACL=MK12	NACL	MK12	-0.06	0	0.26
16	NACL=MK12	NACL	MKO3	-0.06	0	0.26
17	NACL=MK12	NACL	STAT5	-0.06	0	0.26
18	REBIF=JAK1	REBIF	STAT1	-0.05	1	0.58
19	TAK1=MK12	IL1A	HSPB1	-0.15	1	0.79
20	TAK1=MK12	IL1A	MK12	-0.15	1	0.79
21	TAK1=MK12	IL1A	MKO3	-0.15	1	0.79
22	TAK1=MK12	IL1A	STAT5	-0.15	1	0.79
23	TAK1=MK12	LPS	HSPB1	-0.15	1	0.79
24	TAK1=MK12	LPS	MK12	-0.15	1	0.79
25	TAK1=MK12	LPS	MKO3	-0.15	1	0.79
26	TAK1=MK12	LPS	STAT5	-0.15	1	0.79
27	TAK1=MK12	POLYIC	HSPB1	-0.15	1	0.79
28	TAK1=MK12	POLYIC	MK12	-0.15	1	0.79
29	TAK1=MK12	POLYIC	MKO3	-0.15	1	0.79
30	TAK1=MK12	POLYIC	STAT5	-0.15	1	0.79

References Table S4

[1] Patricia A Zipfel, Weiguo Zhang, Marisol Quiroz, and Ann Marie Pendergast. Requirement for Abl kinases in T cell receptor signaling. *Current biology : CB*, 14(14):1222–31, jul 2004.

[2] Masahito Hanada, Jianhua Feng, and Brian A Hemmings. Structure, regulation and function of PKB/AKT–a major therapeutic target. *Biochimica et biophysica acta*, 1697(1-2):3–16, mar 2004.

[3] K Du and M Montminy. CREB is a regulatory target for the protein kinase Akt/PKB. *The Journal of biological chemistry*, 273(49):32377–9, dec 1998.

[4] A Brunet, J Park, H Tran, L S Hu, B A Hemmings, and M E Greenberg. Protein kinase SGK mediates survival signals by phosphorylating the forkhead transcription factor FKHRL1 (FOXO3a). *Molecular and cellular biology*, 21(3):952–65, feb 2001.

[5] Jiyong Liang and Joyce M Slingerland. Multiple roles of the PI3K/PKB (Akt) pathway in cell cycle progression. *Cell cycle (Georgetown, Tex.)*, 2(4):339–45, jan 2003.

[6] Han C Dan, Matthew J Cooper, Patricia C Cogswell, Joseph A Duncan, Jenny P-Y Ting, and Albert S Baldwin. Akt-dependent regulation of NF-{kappa}B is controlled by mTOR and Raptor in association with IKK. *Genes & development*, 22(11):1490–500, jun 2008.

[7] Karin Moelling, Karen Schad, Magnus Bosse, Sven Zimmermann, and Marc Schweneker. Regulation of Raf-Akt Cross-talk. *The Journal of biological chemistry*, 277(34):31099–106, aug 2002.

[8] Isabelle Bekeredjian-Ding, Anne Doster, Martin Schiller, Petra Heyder, Hanns-Martin Lorenz, Burkhart Schraven, Ursula Bommhardt, and Klaus Heeg. TLR9-activating DNA up-regulates ZAP70 via sustained PKB induction in IgM+ B cells. *Journal of immunology (Baltimore, Md. : 1950)*, 181(12):8267–77, dec 2008.

[9] Queenie Lai Kwan Lam, Bo-Jian Zheng, Dong-Yan Jin, Xuetao Cao, and Liwei Lu. Leptin induces CD40 expression through the activation of Akt in murine dendritic cells. *The Journal of biological chemistry*, 282(38):27587–97, sep 2007.

[10] Tilo Beyer, Mandy Busse, Kroum Hristov, Slavyana Gurbiel, Michal Smida, Utz-Uwe Haus, Kathrin Ballerstein, Frank Pfeuffer, Robert Weismantel, Burkhart Schraven, and Jonathan A Lindquist. Integrating signals from the T-cell receptor and the interleukin-2 receptor. *PLoS computational biology*, 7(8):e1002121, aug 2011.

[11] Viveka Mayya, Deborah H Lundgren, Sun-Il Hwang, Karim Rezaul, Linfeng Wu, Jimmy K Eng, Vladimir Rodionov, and David K Han. Quantitative phosphoproteomic analysis of T cell receptor signaling reveals system-wide modulation of protein-protein interactions. *Science signaling*, 2(84):ra46, jan 2009.

[12] J. Zha, H. Harada, K. Osipov, J. Jockel, G. Waksman, and S. J. Korsmeyer. BH3 Domain of BAD Is Required for Heterodimerization with BCL-XL and Pro-apoptotic Activity. *Journal of Biological Chemistry*, 272(39):24101–24104, sep 1997.

[13] Elizabeth Yang, Jiping Zha, Jennifer Jockel, Lawrence H Boise, Craig B Thompson, and Stanley J Korsmeyer. Bad, a heterodimeric partner for Bcl-xL and Bcl-2, displaces bax and promotes cell death. *Cell*, 80(2):285–291, jan 1995.

[14] R Weil and A Israël. Deciphering the pathway from the TCR to NF-kappaB. *Cell death and differentiation*, 13(5):826–33, may 2006.

[15] Margot Thome. CARMA1, BCL-10 and MALT1 in lymphocyte development and activation. *Nature reviews. Immunology*, 4(5):348–59, may 2004.

[16] Matthew S Hayden and Sankar Ghosh. Signaling to NF-kappaB. *Genes & development*, 18(18):2195–224, sep 2004.

[17] M O Hengartner. The biochemistry of apoptosis. *Nature*, 407(6805):770–6, oct 2000.

[18] A J Levine, W Hu, and Z Feng. The P53 pathway: what questions remain to be explored? *Cell death and differentiation*, 13(6):1027–36, jun 2006.

[19] Michael R Gold. To make antibodies or not: signaling by the B-cell antigen receptor. *Trends in pharmacological sciences*, 23(7):316–24, jul 2002.

[20] D Soppet, E Escandon, J Maragos, D S Middlemas, S W Reid, J Blair, L E Burton, B R Stanton, D R Kaplan, T Hunter, K Nikolics, and L F Parada. The neurotrophic factors brain-derived neurotrophic factor and neurotrophin-3 are ligands for the trkB tyrosine kinase receptor. *Cell*, 65(5):895–903, may 1991.

[21] P Villoslada, G Vila, V Colafrancesco, B Moreno, B Fernandez-Diez, R Vazquez, I Pertsovskaya, I Zubizarreta, I Pulido-Valdeolivas, J Messeguer, G Vendrell-Navarro, JM Frade, N López-Sánchez, M Teixido, E Giralt, M Masso, JC Dugas, D Leonoudakis, KD Lariosa-Willingham, L Steinman, and A Messeguer. Axonal and myelin neuroprotection by the peptoid bn201 in brain inflammation. *Neurotherapeutics*, 16(3):808–827, 2019.

[22] S Feske, J Giltnane, R Dolmetsch, L M Staudt, and A Rao. Gene regulation mediated by calcium signals in T lymphocytes. *Nature immunology*, 2(4):316–24, apr 2001.

[23] S Matsuda, F Shibasaki, K Takehana, H Mori, E Nishida, and S Koyasu. Two distinct action mechanisms of immunophilin-ligand complexes for the blockade of T-cell activation. *EMBO reports*, 1(5):428–34, nov 2000.

[24] Sandra Ryeom, Rebecca J Greenwald, Arlene H Sharpe, and Frank McKeon. The threshold pattern of calcineurin-dependent gene expression is altered by loss of the endogenous inhibitor calcipressin. *Nature immunology*, 4(9):874–81, sep 2003.

[25] A Kashishian, M Howard, C Loh, W M Gallatin, M F Hoekstra, and Y Lai. AKAP79 inhibits calcineurin through a site distinct from the immunophilin-binding region. *The Journal of biological chemistry*, 273(42):27412–9, oct 1998.

[26] Yanping Huang and Ronald L Wange. T cell receptor signaling: beyond complex complexes. *The Journal of biological chemistry*, 279(28):28827–30, jul 2004.

[27] Fernando Macian. NFAT proteins: key regulators of T-cell development and function. *Nature reviews. Immunology*, 5(6):472–84, jun 2005.

[28] Gerhard Krauss. *Biochemistry of Signal Transduction and Regulation.* John Wiley & Sons, 2006.

[29] K Hughes, S Edin, A Antonsson, and T Grundström. Calmodulin-dependent kinase II mediates T cell receptor/CD3- and phorbol ester-induced activation of IkappaB kinase. *The Journal of biological chemistry*, 276(38):36008–13, sep 2001.

[30] K E Anderson, J Coadwell, L R Stephens, and P T Hawkins. Translocation of PDK-1 to the plasma membrane is important in allowing PDK-1 to activate protein kinase B. *Current biology : CB*, 8(12):684–91, jun 1998.

[31] D D Schlaepfer, M A Broome, and T Hunter. Fibronectin-stimulated signaling from a focal adhesion kinase-c-Src complex: involvement of the Grb2, p130cas, and Nck adaptor proteins. *Molecular and cellular biology*, 17(3):1702–13, mar 1997.

[32] M L Galisteo, I Dikic, A G Batzer, W Y Langdon, and J Schlessinger. Tyrosine phosphorylation of the c-cbl proto-oncogene protein product and association with epidermal growth factor (EGF) receptor upon EGF stimulation. *The Journal of biological chemistry*, 270(35):20242–5, sep 1995.

[33] Gabi Tarcic, Shlomit K Boguslavsky, Jean Wakim, Tai Kiuchi, Angela Liu, Felicia Reinitz, David Nathanson, Takamune Takahashi, Paul S Mischel, Tony Ng, and Yosef Yarden. An unbiased screen identifies DEP-1 tumor suppressor as a phosphatase controlling EGFR endocytosis. *Current biology : CB*, 19(21):1788–98, nov 2009.

[34] K. Kaabeche, J. Lemonnier, S. Le Mee, J. Caverzasio, and P. J. Marie. Cbl-mediated Degradation of Lyn and Fyn Induced by Constitutive Fibroblast Growth Factor Receptor-2 Activation Supports Osteoblast Differentiation. *Journal of Biological Chemistry*, 279(35):36259–36267, jun 2004.

[35] Lei Duan, Alagarsamy Lakku Reddi, Amiya Ghosh, Manjari Dimri, and Hamid Band. The Cbl family and other ubiquitin ligases: destructive forces in control of antigen receptor signaling. *Immunity*, 21(1):7–17, jul 2004.

[36] A. L. Reddi, G. Ying, L. Duan, G. Chen, M. Dimri, P. Douillard, B. J. Druker, M. Naramura, V. Band, and H. Band. Binding of Cbl to a Phospholipase C 1-docking Site on Platelet-derived Growth Factor Receptor beta Provides a Dual Mechanism of Negative Regulation. *Journal of Biological Chemistry*, 282(40):29336–29347, aug 2007.

[37] Piotr Wardega, Carl-Henrik Heldin, and Johan Lennartsson. Mutation of tyrosine residue 857 in the PDGF beta-receptor affects cell proliferation but not migration. *Cellular signalling*, 22(9):1363–8, sep 2010.

[38] D Fang and Y C Liu. Proteolysis-independent regulation of PI3K by Cbl-b-mediated ubiquitination in T cells. *Nature immunology*, 2(9):870–5, sep 2001.

[39] N Rao, M L Lupher, S Ota, K A Reedquist, B J Druker, and H Band. The linker phosphorylation site Tyr292 mediates the negative regulatory effect of Cbl on ZAP-70 in T cells. *Journal of immunology (Baltimore, Md. : 1950)*, 164(9):4616–26, may 2000.

[40] J H Ellis, C Ashman, M N Burden, K E Kilpatrick, M A Morse, and P A Hamblin. GRID: a novel Grb-2-related adapter protein that interacts with the activated T cell costimulatory receptor CD28. *Journal of immunology (Baltimore, Md. : 1950)*, 164(11):5805–14, jun 2000.

[41] K Okkenhaug and R Rottapel. Grb2 forms an inducible protein complex with CD28 through a Src homology 3 domain-proline interaction. *The Journal of biological chemistry*, 273(33):21194–202, aug 1998.

[42] J A Nunès, A Truneh, D Olive, and D A Cantrell. Signal transduction by CD28 costimulatory receptor on T cells. B7-1 and B7-2 regulation of tyrosine kinase adaptor molecules. *The Journal of biological chemistry*, 271(3):1591–8, jan 1996.

[43] A August and B Dupont. CD28 of T lymphocytes associates with phosphatidylinositol 3-kinase. *International immunology*, 6(5):769–74, may 1994.

[44] M Ghiotto-Ragueneau, M Battifora, A Truneh, M D Waterfield, and D Olive. Comparison of CD28-B7.1 and B7.2 functional interaction in resting human T cells: phosphatidylinositol 3-kinase association to CD28 and cytokine production. *European journal of immunology*, 26(1):34–41, jan 1996.

[45] Julio Saez-Rodriguez, Luca Simeoni, Jonathan A Lindquist, Rebecca Hemenway, Ursula Bommhardt, Boerge Arndt, Utz-Uwe Haus, Robert Weismantel, Ernst D Gilles, Steffen Klamt, and Burkhart Schraven. A logical model provides insights into T cell receptor signaling. *PLoS computational biology*, 3(8):e163, aug 2007.

[46] Emil H Palacios and Arthur Weiss. Function of the Src-family kinases, Lck and Fyn, in T-cell development and activation. *Oncogene*, 23(48):7990–8000, oct 2004.

[47] Dominik Filipp, Bernadine L Leung, Jenny Zhang, André Veillette, and Michael Julius. Enrichment of lck in lipid rafts regulates colocalized fyn activation and the initiation of proximal signals through TCR alpha beta. *Journal of immunology (Baltimore, Md. : 1950)*, 172(7):4266–74, apr 2004.

[48] G R Fanger, N L Johnson, and G L Johnson. MEK kinases are regulated by EGF and selectively interact with Rac/Cdc42. *The EMBO journal*, 16(16):4961–72, aug 1997.

[49] S Kaga, S Ragg, K A Rogers, and A Ochi. Activation of p21-CDC42/Rac-activated kinases by CD28 signaling: p21-activated kinase (PAK) and MEK kinase 1 (MEKK1) may mediate the interplay between CD3 and CD28 signals. *Journal of immunology (Baltimore, Md. : 1950)*, 160(9):4182–9, may 1998.

[50] C S Hill, J Wynne, and R Treisman. The Rho family GTPases RhoA, Rac1, and CDC42Hs regulate transcriptional activation by SRF. *Cell*, 81(7):1159–70, jun 1995.

[51] Guanghu Wang, Jeane Silva, Kannan Krishnamurthy, Eric Tran, Brian G Condie, and Erhard Bieberich. Direct binding to ceramide activates protein kinase Czeta before the formation of a pro-apoptotic complex with PAR-4 in differentiating stem cells. *The Journal of biological chemistry*, 280(28):26415–24, jul 2005.

[52] S de Petris. Concanavalin A receptors, immunoglobulins, and theta antigen of the lymphocyte surface. Interactions with concanavalin A and with Cytoplasmic structures. *The Journal of cell biology*, 65(1):123–46, apr 1975.

[53] P Cuatrecasas. Interaction of concanavalin A and wheat germ agglutinin with the insulin receptor of fat cells and liver. *The Journal of biological chemistry*, 248(10):3528–34, may 1973.

[54] S F Amato, K Nakajima, T Hirano, and T C Chiles. Transcriptional regulation of the junB gene in B lymphocytes: role of protein kinase A and a membrane Ig-regulated protein phosphatase. *Journal of immunology (Baltimore, Md. : 1950)*, 159(10):4676–85, nov 1997.

[55] Kathleen M Campbell and Kevin J Lumb. Structurally distinct modes of recognition of the KIX domain of CBP by Jun and CREB. *Biochemistry*, 41(47):13956–64, nov 2002.

[56] Pier Paolo Di Fiore. Signal transduction: life on Mars, cellularly speaking. *Nature*, 424(6949):624–5, aug 2003.

[57] Trever G Bivona, Ignacio Pérez De Castro, Ian M Ahearn, Theresa M Grana, Vi K Chiu, Peter J Lockyer, Peter J Cullen, Angel Pellicer, Adrienne D Cox, and Mark R Philips. Phospholipase Cgamma activates Ras on the Golgi apparatus by means of RasGRP1. *Nature*, 424(6949):694–8, aug 2003.

[58] Ki-Young Lee, Fulvio D'Acquisto, Matthew S Hayden, Jae-Hyuck Shim, and Sankar Ghosh. PDK1 nucleates T cell receptor-induced signaling complex for NF-kappaB activation. *Science (New York, N.Y.)*, 308(5718):114–8, apr 2005.

[59] Matthew K Topham. Signaling roles of diacylglycerol kinases. *Journal of cellular biochemistry*, 97(3):474–84, feb 2006.

[60] Ralf Gold, Ludwig Kappos, Douglas L Arnold, Amit Bar-Or, Gavin Giovannoni, Krzysztof Selmaj, Carlo Tornatore, Marianne T Sweetser, Minhua Yang, Sarah I Sheikh, and Katherine T Dawson. Placebo-controlled phase 3 study of oral BG-12 for relapsing multiple sclerosis. *The New England journal of medicine*, 367(12):1098–107, sep 2012.

[61] M Michailidou, IN Melas, DE Messinis, S Klamt, LG Alexopoulos, FN Kolisis, and H Loutrari. Network-Based Analysis of Nutraceuticals in Human Hepatocellular Carcinomas Reveals Mechanisms of Chemopreventive Action. *CPT: Pharmacometrics & Systems Pharmacology*, 4(6):350–361, jun 2015.

[62] Subash C Gupta, Ji Hye Kim, Sahdeo Prasad, and Bharat B Aggarwal. Regulation of survival, proliferation, invasion, angiogenesis, and metastasis of tumor cells through modulation of inflammatory pathways by nutraceuticals. *Cancer metastasis reviews*, 29(3):405–34, sep 2010.

[63] Glenn S Van Aller, Jeff D Carson, Wei Tang, Hao Peng, Lin Zhao, Robert A Copeland, Peter J Tummino, and Lusong Luo. Epigallocatechin gallate (EGCG), a major component of green tea, is a dual phosphoinositide-3-kinase/mTOR inhibitor. *Biochemical and biophysical research communications*, 406(2):194–9, mar 2011.

[64] Masahito Shimizu, Atsuko Deguchi, Yukihiko Hara, Hisataka Moriwaki, and I Bernard Weinstein. EGCG inhibits activation of the insulin-like growth factor-1 receptor in human colon cancer cells. *Biochemical and biophysical research communications*, 334(3):947–53, sep 2005.

[65] Hanyong Chen, Ke Yao, Xiaoyu Chang, Jung-Hyun Shim, Hong-Gyum Kim, Margarita Malakhova, Dong-Joon Kim, Ann M. Bode, Zigang Dong, D Mereles, W Hunstein, BN Singh, S Shankar, RK Srivastava, DJ Yang, LS Hwang, SA Milligan, P Burke, DT Coleman, RL Bigelow, JJ Steffan, JL Carroll, TA Zykova, Y Zhang, F Zhu, AM Bode, Z Dong, JH Shim, HS Choi, A Pugliese, SY Lee,

JI Chae, BY Choi, Z Dong, W Ma, C Huang, CS Yang, Y Suzuki, M Isemura, S Ermakova, BY Choi, HS Choi, BS Kang, AM Bode, Z Dong, SP Ermakova, BS Kang, BY Choi, HS Choi, TF Schuster, WY Ma, JH Shim, ZY Su, JI Chae, DJ Kim, F Zhu, WY Ma, M Leone, D Zhai, S Sareth, S Kitada, JC Reed, M Pellecchia, H Tachibana, K Koga, Y Fujimura, K Yamada, D Chen, S Pamu, Q Cui, TH Chan, QP Dou, DV Urusova, JH Shim, DJ Kim, SK Jung, TA Zykova, A Carper, M Frodin, CJ Jensen, K Merienne, S Gammeltoft, YY Cho, K Yao, AM Bode, HR Bergen 3rd, BJ Madden, SM Oh, YY Cho, K Yao, HG Kim, BS Kang, D Zheng, AM Bode, HM Berman, J Westbrook, Z Feng, G Gilliland, TN Bhat, H Weissig, M Malakhova, I Kurinov, K Liu, D Zheng, I D'Angelo, JH Shim, M Malakhova, V Tereshko, SY Lee, K Yao, YY Cho, A Bode, AC Dar, TK Das, KM Shokat, RL Cagan, U Derewenda, M Artamonov, G Szukalska, D Utepbergenov, N Olekhnovich, HI Parikh, D Utepbergenov, U Derewenda, N Olekhnovich, G Szukalska, B Banerjee, MK Hilinski, P Sassone-Corsi, CA Mizzen, P Cheung, C Crosio, L Monaco, S Jacquot, R Anjum, J Blenis, YY Cho, K Yao, A Pugliese, ML Malakhova, AM Bode, Z Dong, CE Poteet-Smith, JA Smith, DA Lannigan, TA Freed, TW Sturgill, DR Knighton, JH Zheng, LF Ten Eyck, VA Ashford, NH Xuong, SS Taylor, JX Lin, R Spolski, WJ Leonard, K Yao, H Chen, K Liu, A Langfald, G Yang, Y Zhang, Z Dong, AM Bode, L Ding, G Getz, DA Wheeler, ER Mardis, MD McLellan, K Cibulskis, LD Wood, DW Parsons, S Jones, J Lin, T Sjoblom, RJ Leary, CE Geyer, J Forster, D Lindquist, S Chan, CG Romieu, T Pienkowski, KB Kim, R Kefford, AC Pavlick, JR Infante, A Ribas, JA Sosman, PM LoRusso, SS Krishnamurthi, JJ Rinehart, LM Nabell, L Malburg, PB Chapman, KW Lee, AM Bode, Z Dong, A Petrelli, S Giordano, A Grover, SP Katiyar, J Jeyakanthan, VK Dubey, D Sundar, N Okimoto, N Futatsugi, H Fuji, A Suenaga, G Morimoto, R Yanai, KC Hsu, WC Cheng, YF Chen, WC Wang, JM Yang, G Sliwoski, S Kothiwale, J Meiler, EW Lowe, Y Sheng, W Li, F Zhu, K Liu, H Chen, K Yao, T Ota, S Suto, H Katayama, ZB Han, F Suzuki, M Maeda, HS Choi, BY Choi, YY Cho, H Mizuno, BS Kang, and AM Bode. Computational and Biochemical Discovery of RSK2 as a Novel Target for Epigallocatechin Gallate (EGCG). *PLOS ONE*, 10(6):e0130049, jun 2015.

[66] C B Thien and W Y Langdon. Cbl: many adaptations to regulate protein tyrosine kinases. *Nature reviews. Molecular cell biology*, 2(4):294–307, apr 2001.

[67] T Okutani, Y Okabayashi, Y Kido, Y Sugimoto, K Sakaguchi, K Matuoka, T Takenawa, and M Kasuga. Grb2/Ash binds directly to tyrosines 1068 and 1086 and indirectly to tyrosine 1148 of activated human epidermal growth factor receptors in intact cells. *The Journal of biological chemistry*, 269(49):31310–4, dec 1994.

[68] Johann Riedemann, Megumi Takiguchi, Muhammad Sohail, and Valentine M Macaulay. The EGF receptor interacts with the type 1 IGF receptor and regulates its stability. *Biochemical and biophysical research communications*, 355(3):707–14, apr 2007.

[69] Samantha Messina, Franco Onofri, Lucilla Bongiorno-Borbone, Silvia Giovedì, Flavia Valtorta, Jean-Antoine Girault, and Fabio Benfenati. Specific interactions of neuronal focal adhesion kinase isoforms with Src kinases and amphiphysin. *Journal of neurochemistry*, 84(2):253–65, jan 2003.

[70] S T Arold, T S Ulmer, T D Mulhern, J M Werner, J E Ladbury, I D Campbell, and M E Noble. The role of the Src homology 3-Src homology 2 interface in the regulation of Src kinases. *The Journal of biological chemistry*, 276(20):17199–205, may 2001.

[71] Dominique Davidson, Marcin Bakinowski, Matthew L Thomas, Vaclav Horejsi, and André Veillette. Phosphorylation-dependent regulation of T-cell activation by PAG/Cbp, a lipid raft-associated transmembrane adaptor. *Molecular and cellular biology*, 23(6):2017–28, mar 2003.

[72] Dominik Filipp and Michael Julius. Lipid rafts: resolution of the "fyn problem"? *Molecular immunology*, 41(6-7):645–56, jul 2004.

[73] S Yamasaki, K Nishida, M Hibi, M Sakuma, R Shiina, A Takeuchi, H Ohnishi, T Hirano, and T Saito. Docking protein Gab2 is phosphorylated by ZAP-70 and negatively regulates T cell receptor signaling by recruitment of inhibitory molecules. *The Journal of biological chemistry*, 276(48):45175–83, nov 2001.

[74] Sho Yamasaki, Keigo Nishida, Machie Sakuma, Donna Berry, C Jane McGlade, Toshio Hirano, and Takashi Saito. Gads/Grb2-mediated association with LAT is critical for the inhibitory function of Gab2 in T cells. *Molecular and cellular biology*, 23(7):2515–29, apr 2003.

[75] Václav Horejsí, Weiguo Zhang, and Burkhart Schraven. Transmembrane adaptor proteins: organizers of immunoreceptor signalling. *Nature reviews. Immunology*, 4(8):603–16, aug 2004.

[76] Mauro Togni, Jon Lindquist, Annegret Gerber, Uwe Kölsch, Andrea Hamm-Baarke, Stefanie Kliche, and Burkhart Schraven. The role of adaptor proteins in lymphocyte activation. *Molecular immunology*, 41(6-7):615–30, jul 2004.

[77] E Genot and D A Cantrell. Ras regulation and function in lymphocytes. *Current opinion in immunology*, 12(3):289–94, jun 2000.

[78] Suzanne Mandala, Richard Hajdu, James Bergstrom, Elizabeth Quackenbush, Jenny Xie, James Milligan, Rosemary Thornton, Gan-Ju Shei, Deborah Card, CarolAnn Keohane, Mark Rosenbach, Jeffrey Hale, Christopher L Lynch, Kathleen Rupprecht, William Parsons, and Hugh Rosen. Alteration of lymphocyte trafficking by sphingosine-1-phosphate receptor agonists. *Science (New York, N.Y.)*, 296(5566):346–9, apr 2002.

[79] P C Heinrich, I Behrmann, G Müller-Newen, F Schaper, and L Graeve. Interleukin-6-type cytokine signalling through the gp130/Jak/STAT pathway. *The Biochemical journal*, 334 (Pt 2:297–314, sep 1998.

[80] Peter J Murray. The JAK-STAT signaling pathway: input and output integration. *Journal of immunology (Baltimore, Md. : 1950)*, 178(5):2623–9, mar 2007.

[81] F Schaper, C Gendo, M Eck, J Schmitz, C Grimm, D Anhuf, I M Kerr, and P C Heinrich. Activation of the protein tyrosine phosphatase SHP2 via the interleukin-6 signal transducing receptor protein gp130 requires tyrosine kinase Jak1 and limits acute-phase protein expression. *The Biochemical journal*, 335 (Pt 3:557–65, nov 1998.

[82] L Buday, S E Egan, P Rodriguez Viciana, D A Cantrell, and J Downward. A complex of Grb2 adaptor protein, Sos exchange factor, and a 36-kDa membrane-bound tyrosine phosphoprotein is implicated in ras activation in T cells. *The Journal of biological chemistry*, 269(12):9019–23, mar 1994.

[83] C R Beals, C M Sheridan, C W Turck, P Gardner, and G R Crabtree. Nuclear export of NF-ATc enhanced by glycogen synthase kinase-3. *Science (New York, N.Y.)*, 275(5308):1930–4, mar 1997.

[84] Almut Schulze and Adrian L Harris. How cancer metabolism is tuned for proliferation and vulnerable to disruption. *Nature*, 491(7424):364–73, nov 2012.

[85] Lorne J Hofseth, S Perwez Hussain, and Curtis C Harris. p53: 25 years after its discovery. *Trends in pharmacological sciences*, 25(4):177–81, apr 2004.

[86] S Etienne-Manneville, J B Manneville, P Adamson, B Wilbourn, J Greenwood, and P O Couraud. ICAM-1-coupled cytoskeletal rearrangements and transendothelial lymphocyte migration involve intracellular calcium signaling in brain endothelial cell lines. *Journal of immunology (Baltimore, Md. : 1950)*, 165(6):3375–83, sep 2000.

[87] Inna Pertsovskaya, Elena Abad, Núria Domedel-Puig, Jordi Garcia-Ojalvo, and Pablo Villoslada. Transient oscillatory dynamics of interferon beta signaling in macrophages. *BMC systems biology*, 7:59, jan 2013.

[88] Axel Weber, Peter Wasiliew, and Michael Kracht. Interleukin-1 (IL-1) pathway. *Science signaling*, 3(105):cm1, jan 2010.

[89] Ann Kelly-Welch, Erica M Hanson, and Achsah D Keegan. Interleukin-4 (IL-4) pathway. *Science's STKE : signal transduction knowledge environment*, 2005(293):cm9, jul 2005.

[90] T Hirano. Interleukin 6 and its receptor: ten years later. *International reviews of immunology*, 16(3-4):249–84, jan 1998.

[91] A R Saltiel and C R Kahn. Insulin signalling and the regulation of glucose and lipid metabolism. *Nature*, 414(6865):799–806, dec 2001.

[92] T O Chan, S E Rittenhouse, and P N Tsichlis. AKT/PKB and other D3 phosphoinositide-regulated kinases: kinase activation by phosphoinositide-dependent phosphorylation. *Annual review of biochemistry*, 68:965–1014, jan 1999.

[93] M J Czar, J Debnath, E M Schaeffer, C M Lewis, and P L Schwartzberg. Biochemical and genetic analyses of the Tec kinases Itk and Rlk/Txk. *Biochemical Society transactions*, 29(Pt 6):863–7, nov 2001.

[94] Barbara L Rellahan, Laurie J Graham, Alexander Y Tysgankov, Karen E DeBell, Maria-Concetta Veri, Cristiana Noviello, and Ezio Bonvini. A dynamic constitutive and inducible binding of c-Cbl by PLCgamma1 SH3 and SH2 domains (negatively) regulates antigen receptor-induced PLCgamma1 activation in lymphocytes. *Experimental cell research*, 289(1):184–94, sep 2003.

[95] T Kisseleva, S Bhattacharya, J Braunstein, and C W Schindler. Signaling through the JAK/STAT pathway, recent advances and future challenges. *Gene*, 285(1-2):1–24, feb 2002.

[96] Haojie Huang and Donald J Tindall. Dynamic FoxO transcription factors. *Journal of cell science*, 120(Pt 15):2479–87, aug 2007.

[97] Fan Pan, Anthony R Means, and Jun O Liu. Calmodulin-dependent protein kinase IV regulates nuclear export of Cabin1 during T-cell activation. *The EMBO journal*, 24(12):2104–13, jun 2005.

[98] Jonathan A Lindquist, Luca Simeoni, and Burkhart Schraven. Transmembrane adapters: attractants for cytoplasmic effectors. *Immunological reviews*, 191:165–82, feb 2003.

[99] J Liou, F Kiefer, A Dang, A Hashimoto, M H Cobb, T Kurosaki, and A Weiss. HPK1 is activated by lymphocyte antigen receptors and negatively regulates AP-1. *Immunity*, 12(4):399–408, apr 2000.

[100] W Zhang, J Sloan-Lancaster, J Kitchen, R P Trible, and L E Samelson. LAT: the ZAP-70 tyrosine kinase substrate that links T cell receptor to cellular activation. *Cell*, 92(1):83–92, jan 1998.

[101] Xiujuan Qu, Keiko Kawauchi-Kamata, S M Shahjahan Miah, Tomoko Hatani, Hirohei Yamamura, and Kiyonao Sada. Tyrosine phosphorylation of adaptor protein 3BP2 induces T cell receptor-mediated activation of transcription factor. *Biochemistry*, 44(10):3891–8, mar 2005.

[102] Rose Zamoyska, Albert Basson, Andrew Filby, Giuseppe Legname, Matthew Lovatt, and Benedict Seddon. The influence of the src-family kinases, Lck and Fyn, on T cell differentiation, survival and activation. *Immunological reviews*, 191:107–18, feb 2003.

[103] Jonathan A Deane and David A Fruman. Phosphoinositide 3-kinase: diverse roles in immune cell activation. *Annual review of immunology*, 22:563–98, jan 2004.

[104] X Shan, M J Czar, S C Bunnell, P Liu, Y Liu, P L Schwartzberg, and R L Wange. Deficiency of PTEN in Jurkat T cells causes constitutive localization of Itk to the plasma membrane and hyperresponsiveness to CD3 stimulation. *Molecular and cellular biology*, 20(18):6945–57, sep 2000.

[105] Emily M L Chastain and Stephen D Miller. Molecular mimicry as an inducing trigger for CNS autoimmune demyelinating disease. *Immunological reviews*, 245(1):227–38, jan 2012.

[106] H Enslen, J Raingeaud, and R J Davis. Selective activation of p38 mitogen-activated protein (MAP) kinase isoforms by the MAP kinase kinases MKK3 and MKK6. *The Journal of biological chemistry*, 273(3):1741–8, jan 1998.

[107] Y Fleming, C G Armstrong, N Morrice, A Paterson, M Goedert, and P Cohen. Synergistic activation of stress-activated protein kinase 1/c-Jun N-terminal kinase (SAPK1/JNK) isoforms by mitogen-activated protein kinase kinase 4 (MKK4) and MKK7. *The Biochemical journal*, 352 Pt 1:145–54, nov 2000.

[108] J Raingeaud, A J Whitmarsh, T Barrett, B Dérijard, and R J Davis. MKK3- and MKK6-regulated gene expression is mediated by the p38 mitogen-activated protein kinase signal transduction pathway. *Molecular and cellular biology*, 16(3):1247–55, mar 1996.

[109] M Yan, T Dai, J C Deak, J M Kyriakis, L I Zon, J R Woodgett, and D J Templeton. Activation of stress-activated protein kinase by MEKK1 phosphorylation of its activator SEK1. *Nature*, 372(6508):798–800, jan 1994.

[110] Z Guan, S Y Buckman, A P Pentland, D J Templeton, and A R Morrison. Induction of cyclooxygenase-2 by the activated MEKK1 –> SEK1/MKK4 –> p38 mitogen-activated protein kinase pathway. *The Journal of biological chemistry*, 273(21):12901–8, may 1998.

[111] L A Tibbles, Y L Ing, F Kiefer, J Chan, N Iscove, J R Woodgett, and N J Lassam. MLK-3 activates the SAPK/JNK and p38/RK pathways via SEK1 and MKK3/6. *The EMBO journal*, 15(24):7026–35, dec 1996.

[112] M C Hu, W R Qiu, X Wang, C F Meyer, and T H Tan. Human HPK1, a novel human hematopoietic progenitor kinase that activates the JNK/SAPK kinase cascade. *Genes & development*, 10(18):2251–64, sep 1996.

[113] Tyler Zarubin and HAN Jiahuai. Activation and signaling of the p38 map kinase pathway. *Cell research*, 15(1):11–18, 2005.

[114] R J Davis. Signal transduction by the JNK group of MAP kinases. *Cell*, 103(2):239–52, oct 2000.

[115] Maria Deak, Andrew D Clifton, John M Lucocq, and Dario R Alessi. Mitogen-and stress-activated protein kinase-1 (msk1) is directly activated by mapk and sapk2/p38, and may mediate activation of creb. *The EMBO journal*, 17(15):4426–4441, 1998.

[116] Rachida S BelAiba, Talija Djordjevic, Steve Bonello, Ferruh Artunc, Florian Lang, John Hess, and Agnes Görlach. The serum- and glucocorticoid-inducible kinase Sgk-1 is involved in pulmonary vascular remodeling: role in redox-sensitive regulation of tissue factor by thrombin. *Circulation research*, 98(6):828–36, mar 2006.

[117] Angela Clerk, Timothy J Kemp, Joanne G Harrison, Anthony J Mullen, Paul J R Barton, and Peter H Sugden. Up-regulation of c-jun mRNA in cardiac myocytes requires the extracellular signal-regulated kinase cascade, but c-Jun N-terminal kinases are required for efficient up-regulation of c-Jun protein. *The Biochemical journal*, 368(Pt 1):101–10, nov 2002.

[118] M Frödin and S Gammeltoft. Role and regulation of 90 kDa ribosomal S6 kinase (RSK) in signal transduction. *Molecular and cellular endocrinology*, 151(1-2):65–77, may 1999.

[119] P Peraldi, Z Zhao, C Filloux, E H Fischer, and E Van Obberghen. Protein-tyrosine-phosphatase 2C is phosphorylated and inhibited by 44-kDa mitogen-activated protein kinase. *Proceedings of the National Academy of Sciences of the United States of America*, 91(11):5002–6, may 1994.

[120] Ynes A Helou, Vinh Nguyen, Samantha P Beik, and Arthur R Salomon. Erk positive feedback regulates a widespread network of tyrosine phosphorylation sites across canonical t cell signaling and actin cytoskeletal proteins in jurkat t cells. *PLoS One*, 8(7):e69641, 2013.

[121] Taro Kawai and Shizuo Akira. The role of pattern-recognition receptors in innate immunity: update on Toll-like receptors. *Nature immunology*, 11(5):373–84, may 2010.

[122] Markus Kleinewietfeld, Arndt Manzel, Jens Titze, Heda Kvakan, Nir Yosef, Ralf A Linker, Dominik N Muller, and David A Hafler. Sodium chloride drives autoimmune disease by the induction of pathogenic TH17 cells. *Nature*, 496(7446):518–22, apr 2013.

[123] Y Katoh, K Itoh, E Yoshida, M Miyagishi, A Fukamizu, and M Yamamoto. Two domains of Nrf2 cooperatively bind CBP, a CREB binding protein, and synergistically activate transcription. *Genes to cells : devoted to molecular & cellular mechanisms*, 6(10):857–68, oct 2001.

[124] R Venugopal and A K Jaiswal. Nrf2 and Nrf1 in association with Jun proteins regulate antioxidant response element-mediated expression and coordinated induction of genes encoding detoxifying enzymes. *Oncogene*, 17(24):3145–56, dec 1998.

[125] Jonathan M Maher, Matthew Z Dieter, Lauren M Aleksunes, Angela L Slitt, Grace Guo, Yuji Tanaka, George L Scheffer, Jefferson Y Chan, Jose E Manautou, Ying Chen, Timothy P Dalton, Masayuki Yamamoto, and Curtis D Klaassen. Oxidative and electrophilic stress induces multidrug resistance-associated protein transporters via the nuclear factor-E2-related factor-2 transcriptional pathway. *Hepatology (Baltimore, Md.)*, 46(5):1597–610, nov 2007.

[126] Vivek K Gupta, Yuyi You, Veer Bala Gupta, Alexander Klistorner, and Stuart L Graham. TrkB receptor signalling: implications in neurodegenerative, psychiatric and proliferative disorders. *International journal of molecular sciences*, 14(5):10122–42, jan 2013.

[127] Piyajit Watcharasit, Gautam N Bijur, Jaroslaw W Zmijewski, Ling Song, Anna Zmijewska, Xinbin Chen, Gail V W Johnson, and Richard S Jope. Direct, activating interaction between glycogen synthase kinase-3beta and p53 after DNA damage. *Proceedings of the National Academy of Sciences of the United States of America*, 99(12):7951–5, jun 2002.

[128] Harsha S Madapura, Daniel Salamon, Klas G Wiman, Sonia Lain, George Klein, Eva Klein, and Noémi Nagy. p53 contributes to T cell homeostasis through the induction of pro-apoptotic SAP. *Cell cycle (Georgetown, Tex.)*, 11(24):4563–9, dec 2012.

[129] Virginie Lafont, Emmanuelle Astoul, Arian Laurence, Janny Liautard, and Doreen Cantrell. The T cell antigen receptor activates phosphatidylinositol 3-kinase-regulated serine kinases protein kinase B and ribosomal S6 kinase 1. *FEBS Letters*, 486(1):38–42, dec 2000.

[130] Dario R. Alessi, Stephen R. James, C.Peter Downes, Andrew B. Holmes, Piers R.J. Gaffney, Colin B. Reese, and Philip Cohen. Characterization of a 3-phosphoinositide-dependent protein kinase which phosphorylates and activates protein kinase Bα. *Current Biology*, 7(4):261–269, apr 1997.

[131] N Pullen, P B Dennis, M Andjelkovic, A Dufner, S C Kozma, B A Hemmings, and G Thomas. Phosphorylation and activation of p70s6k by PDK1. *Science (New York, N.Y.)*, 279(5351):707–10, jan 1998.

[132] Heather J Hinton, Dario R Alessi, and Doreen A Cantrell. The serine kinase phosphoinositide-dependent kinase 1 (PDK1) regulates T cell development. *Nature immunology*, 5(5):539–45, may 2004.

[133] R G Gronwald, F J Grant, B A Haldeman, C E Hart, P J O'Hara, F S Hagen, R Ross, D F Bowen-Pope, and M J Murray. Cloning and expression of a cDNA coding for the human platelet-derived growth factor receptor: evidence for more than one receptor class. *Proceedings of the National Academy of Sciences of the United States of America*, 85(10):3435–9, may 1988.

[134] K Yokote, S Mori, K Hansen, J McGlade, T Pawson, C H Heldin, and L Claesson-Welsh. Direct interaction between Shc and the platelet-derived growth factor beta-receptor. *The Journal of biological chemistry*, 269(21):15337–43, may 1994.

[135] Klaus Okkenhaug and Bart Vanhaesebroeck. PI3K in lymphocyte development, differentiation and activation. *Nature reviews. Immunology*, 3(4):317–30, apr 2003.

[136] L E Rameh and L C Cantley. The role of phosphoinositide 3-kinase lipid products in cell function. *The Journal of biological chemistry*, 274(13):8347–50, mar 1999.

[137] A Balendran, R Currie, C G Armstrong, J Avruch, and D R Alessi. Evidence that 3-phosphoinositide-dependent protein kinase-1 mediates phosphorylation of p70 S6 kinase in vivo at Thr-412 as well as Thr-252. *The Journal of biological chemistry*, 274(52):37400–6, dec 1999.

[138] Gang Song, Gaoliang Ouyang, and Shideng Bao. The activation of Akt/PKB signaling pathway and cell survival. *Journal of cellular and molecular medicine*, 9(1):59–71, jan 2005.

[139] Alfonso Mora, David Komander, Daan M F van Aalten, and Dario R Alessi. PDK1, the master regulator of AGC kinase signal transduction. *Seminars in cell & developmental biology*, 15(2):161–70, apr 2004.

[140] A Khoshnan, D Bae, C A Tindell, and A E Nel. The physical association of protein kinase C theta with a lipid raft-associated inhibitor of kappa B factor kinase (IKK) complex plays a role in the activation of the NF-kappa B cascade by TCR and CD28. *Journal of immunology (Baltimore, Md. : 1950)*, 165(12):6933–40, dec 2000.

[141] Malika Bsibsi, Anita Nomden, Johannes M van Noort, and Wia Baron. Toll-like receptors 2 and 3 agonists differentially affect oligodendrocyte survival, differentiation, and myelin membrane formation. *Journal of neuroscience research*, 90(2):388–98, feb 2012.

[142] H Kim and H Baumann. Dual signaling role of the protein tyrosine phosphatase SHP-2 in regulating expression of acute-phase plasma proteins by interleukin-6 cytokine receptors in hepatic cells. *Molecular and cellular biology*, 19(8):5326–38, aug 1999.

[143] H Teramoto, O A Coso, H Miyata, T Igishi, T Miki, and J S Gutkind. Signaling from the small GTP-binding proteins Rac1 and Cdc42 to the c-Jun N-terminal kinase/stress-activated protein kinase pathway. A role for mixed lineage kinase 3/protein-tyrosine kinase 1, a novel member of the mixed lineage kinase family. *The Journal of biological chemistry*, 271(44):27225–8, nov 1996.

[144] R A Franklin, A Tordai, H Patel, A M Gardner, G L Johnson, and E W Gelfand. Ligation of the T cell receptor complex results in activation of the Ras/Raf-1/MEK/MAPK cascade in human T lymphocytes. *The Journal of clinical investigation*, 93(5):2134–40, may 1994.

[145] Rama Soundararajan, Jian Wang, Daniël Melters, and David Pearce. Glucocorticoid-induced Leucine zipper 1 stimulates the epithelial sodium channel by regulating serum- and glucocorticoid-induced kinase 1 stability and subcellular localization. *The Journal of biological chemistry*, 285(51):39905–13, dec 2010.

[146] K Kawauchi, A H Lazarus, J S Sanghera, G L Man, S L Pelech, and T L Delovitch. Regulation of BCR- and PKC/Ca(2+)-mediated activation of the Raf1/MEK/MAPK pathway by protein-tyrosine kinase and -tyrosine phosphatase activities. *Molecular immunology*, 33(3):287–96, feb 1996.

[147] Huizhi Wang, Jonathan Brown, Carlos A Garcia, Yunan Tang, Manjunatha R Benakanakere, Terrance Greenway, Pascale Alard, Denis F Kinane, and Michael Martin. The role of glycogen synthase kinase 3 in regulating IFN-β-mediated IL-10 production. *Journal of immunology (Baltimore, Md. : 1950)*, 186(2):675–84, jan 2011.

[148] Paul Dent, Adly Yacoub, Paul B Fisher, Michael P Hagan, and Steven Grant. Mapk pathways in radiation responses. *Oncogene*, 22(37):5885–5896, 2003.

[149] M J Lee, J R Van Brocklyn, S Thangada, C H Liu, A R Hand, R Menzeleev, S Spiegel, and T Hla. Sphingosine-1-phosphate as a ligand for the G protein-coupled receptor EDG-1. *Science (New York, N.Y.)*, 279(5356):1552–5, mar 1998.

[150] Jerold Chun and Volker Brinkmann. A mechanistically novel, first oral therapy for multiple sclerosis: the development of fingolimod (fty720, gilenya). *Discovery medicine*, 12(64):213, 2011.

[151] Samuel David and Robert G Kalb. Serum/glucocorticoid-inducible kinase can phosphorylate the cyclic AMP response element binding protein, CREB. *FEBS letters*, 579(6):1534–8, feb 2005.

[152] Fakhera Feroze-Zaidi, Luca Fusi, Masashi Takano, Jenny Higham, Madhuri S Salker, Tomoko Goto, Seby Edassery, Karin Klingel, Krishna Murthy Boini, Monica Palmada, Rick Kamps, Patrick G Groothuis, Eric W-F Lam, Stephen K Smith, Florian Lang, Andrew M Sharkey, and Jan J Brosens. Role and regulation of the serum- and glucocorticoid-regulated kinase 1 in fertile and infertile human endometrium. *Endocrinology*, 148(10):5020–9, oct 2007.

[153] Takuma Aoyama, Takashi Matsui, Mikhail Novikov, Jongsun Park, Brian Hemmings, and Anthony Rosenzweig. Serum and glucocorticoid-responsive kinase-1 regulates cardiomyocyte survival and hypertrophic response. *Circulation*, 111(13):1652–9, apr 2005.

[154] Liping Zhang, Ruwen Cui, Xiaodong Cheng, and Jie Du. Antiapoptotic effect of serum and glucocorticoid-inducible protein kinase is mediated by novel mechanism activating I{kappa}B kinase. *Cancer research*, 65(2):457–64, jan 2005.

[155] Minho Won, Kyeong Ah Park, Hee Sun Byun, Young-Rae Kim, Byung Lyul Choi, Jang Hee Hong, Jongsun Park, Jeong Ho Seok, Young-Ho Lee, Chung-Hyun Cho, In Sang Song, Yong Kyung Kim, Han-Ming Shen, and Gang Min Hur. Protein kinase SGK1 enhances MEK/ERK complex formation through the phosphorylation of ERK2: implication for the positive regulatory role of SGK1 on the ERK function during liver regeneration. *Journal of hepatology*, 51(1):67–76, jul 2009.

[156] R. M. Biondi. The PIF-binding pocket in PDK1 is essential for activation of S6K and SGK, but not PKB. *The EMBO Journal*, 20(16):4380–4390, aug 2001.

[157] Shaheen Zakaria, Timothy S Gomez, Doris N Savoy, Simon McAdam, Martin Turner, Robert T Abraham, and Daniel D Billadeau. Differential regulation of TCR-mediated gene transcription by Vav family members. *The Journal of experimental medicine*, 199(3):429–34, feb 2004.

[158] Johannes Brockdorff, Scott Williams, Clément Couture, and Tomas Mustelin. Dephosphorylation of zap-70 and inhibition of t cell activation by activated shp1. *European journal of immunology*, 29(8):2539–2550, 1999.

[159] Eun Kyung Shim, Seung Hee Jung, and Jong Ran Lee. Role of two adaptor molecules SLP-76 and LAT in the PI3K signaling pathway in activated T cells. *Journal of immunology (Baltimore, Md. : 1950)*, 186(5):2926–35, mar 2011.

[160] X P Chen, J A Losman, and P Rothman. SOCS proteins, regulators of intracellular signaling. *Immunity*, 13(3):287–90, sep 2000.

[161] Ling Zhang, Donna B Badgwell, Jack J Bevers, Karni Schlessinger, Peter J Murray, David E Levy, and Stephanie S Watowich. IL-6 signaling via the STAT3/SOCS3 pathway: functional analysis of the conserved STAT3 N-domain. *Molecular and cellular biochemistry*, 288(1-2):179–89, aug 2006.

[162] Edward Yang, Lorena Lerner, Daniel Besser, and James E Darnell. Independent and cooperative activation of chromosomal c-fos promoter by STAT3. *The Journal of biological chemistry*, 278(18):15794–9, may 2003.

[163] Azzam A Maghazachi. Insights into seven and single transmembrane-spanning domain receptors and their signaling pathways in human natural killer cells. *Pharmacological reviews*, 57(3):339–57, sep 2005.

[164] Ioannis Tassiulas, Xiaoyu Hu, Hao Ho, Yogita Kashyap, Paul Paik, Yongmei Hu, Clifford A Lowell, and Lionel B Ivashkiv. Amplification of IFN-alpha-induced STAT1 activation and inflammatory function by Syk and ITAM-containing adaptors. *Nature immunology*, 5(11):1181–9, nov 2004.

[165] M A Sanjuán, D R Jones, M Izquierdo, and I Mérida. Role of diacylglycerol kinase alpha in the attenuation of receptor signaling. *The Journal of cell biology*, 153(1):207–20, apr 2001.

[166] S K Manna and B B Aggarwal. Immunosuppressive leflunomide metabolite (A77 1726) blocks TNF-dependent nuclear factor-kappa B activation and gene expression. *Journal of immunology (Baltimore, Md. : 1950)*, 162(4):2095–102, feb 1999.

[167] Petrus J W Naudé, Johan A den Boer, Paul G M Luiten, and Ulrich L M Eisel. Tumor necrosis factor receptor cross-talk. *The FEBS journal*, 278(6):888–98, apr 2011.

[168] Kim Newton and Vishva M Dixit. Signaling in innate immunity and inflammation. *Cold Spring Harbor perspectives in biology*, 4(3), mar 2012.

[169] F Martinon, N Holler, C Richard, and J Tschopp. Activation of a pro-apoptotic amplification loop through inhibition of NF-kappaB-dependent survival signals by caspase-mediated inactivation of RIP. *FEBS letters*, 468(2-3):134–6, feb 2000.

[170] Martin Villalba, Kun Bi, Junru Hu, Yoav Altman, Paul Bushway, Eric Reits, Jacques Neefjes, Gottfried Baier, Robert T Abraham, and Amnon Altman. Translocation of PKC[theta] in T cells is mediated by a nonconventional, PI3-K- and Vav-dependent pathway, but does not absolutely require phospholipase C. *The Journal of cell biology*, 157(2):253–63, apr 2002.

[171] M. Vidal, C. V. Ramana, and A. S. Dusso. Stat1-Vitamin D Receptor Interactions Antagonize 1,25-Dihydroxyvitamin D Transcriptional Activity and Enhance Stat1-Mediated Transcription. *Molecular and Cellular Biology*, 22(8):2777–2787, apr 2002.

[172] Jesus M Salvador, Paul R Mittelstadt, Galina I Belova, Albert J Fornace, and Jonathan D Ashwell. The autoimmune suppressor Gadd45alpha inhibits the T cell alternative p38 activation pathway. *Nature immunology*, 6(4):396–402, apr 2005.
