# Supplementary material for: Prediction of combination therapies based on topological modeling of the immune signaling network in multiple sclerosis
Source: Genome Med. 2021 Jul 16;13:117. doi: 10.1186/s13073-021-00925-8 (PMC8284018; doi:10.1186/s13073-021-00925-8)
Supplement: Supplementary file 3 — Additional file 3: Supplementary figures S1 to S6. [file 13073_2021_925_MOESM3_ESM.pdf]

Additional file 3.

Supplementary figures S1 to S6

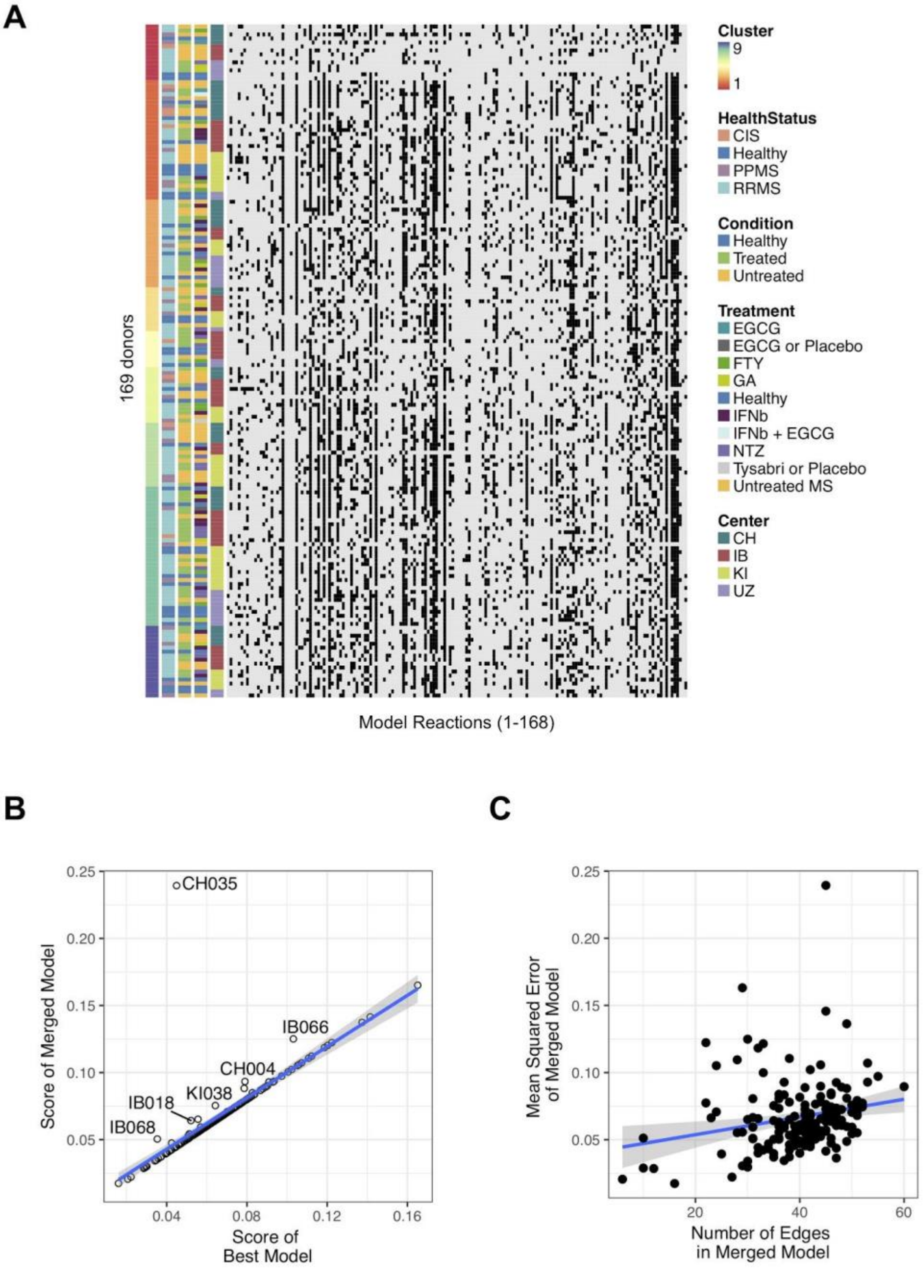
A
B
C
169 donors
Model Reactions (1-168)
Cluster
9
1
HealthStatus
CIS
Healthy
PPMS
RRMS
Condition
Healthy
Treated
Untreated
Treatment
EGCG
EGCG or Placebo
FTY
GA
Healthy
IFNb
IFNb + EGCG
NTZ
Tysabri or Placebo
Untreated MS
Center
CH
IB
KI
UZ
Score of Merged Model
Score of Best Model
CH035
IB066
CH004
KI038
IB018
IB068
0.25
0.20
0.15
0.10
0.05
0.04
0.08
0.12
0.16
Mean Squared Error of Merged Model
Number of Edges in Merged Model
20
40
60

Figure S1. Model quality analysis confirms robustness at the single patient level. A) Signaling network found by modeling for each donor, visualized as a heatmap. Rows: Single donor network. Columns: Signaling activity determined for each interaction by calibrating the PKN shown in Figure S4 after removing the unidentifiable interactions using the phosphoproteomics dataset of each donor. No bias was found due to confounding variables using affinity propagation clustering of the final donor models. The resulting clusters are not enriched for treatment, center, disease subtype or medical condition; **B)** Score of best model found for each patient compared to the final median solution. No new, median models were found to be better than their corresponding best solution, indicating successful optimization; **C)** No relationship was found between model size and performance as quantified by Mean Squared Error between model simulation and data, supporting model quality.

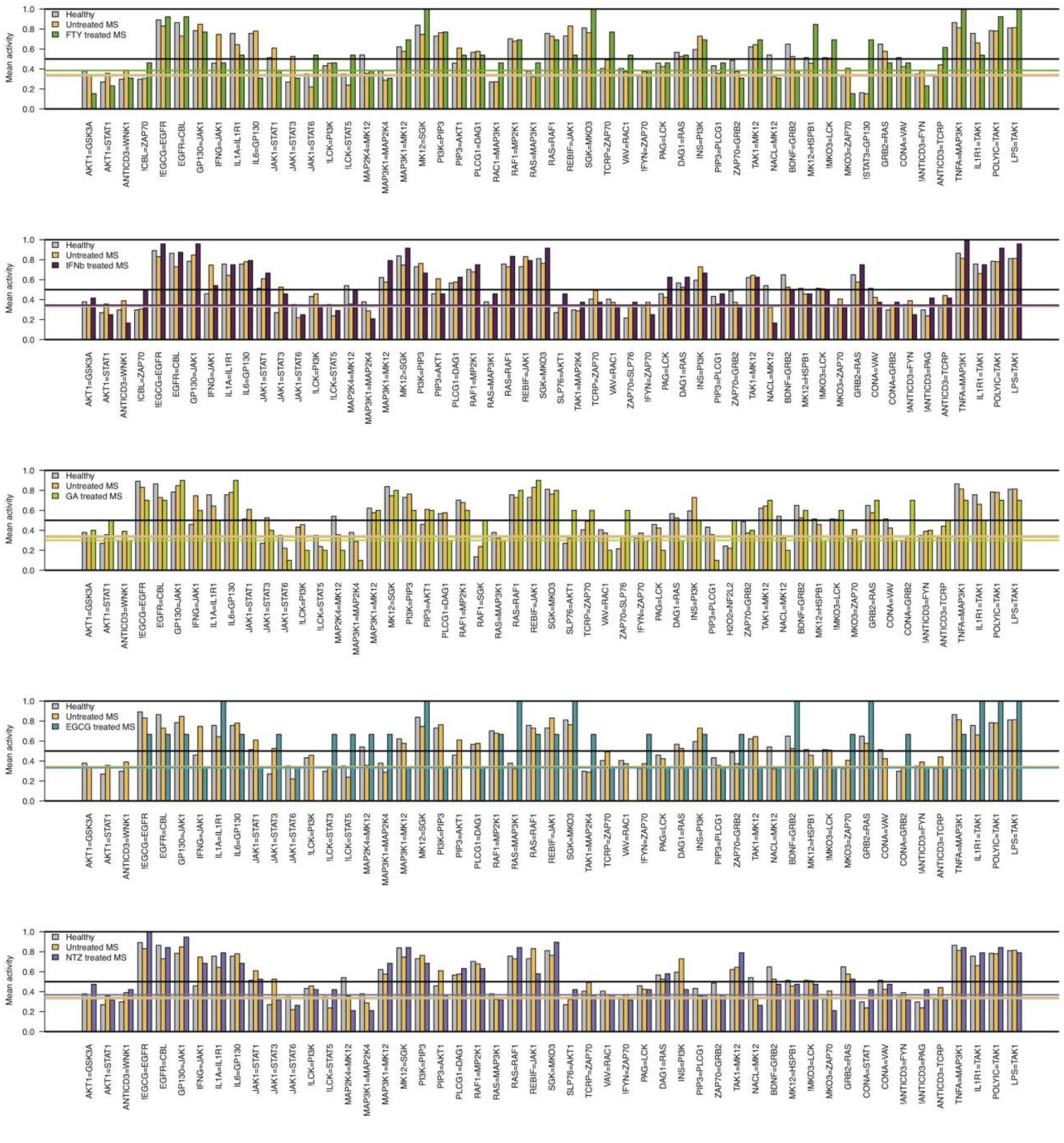

Figure S2. Signaling activity of each interaction of the subgroup networks. Bars represent the signaling activity of each interaction for each donor subgroup, after merging the models for each individual donor by the mean across subgroup donors. Each one of the 5 panels shows the signaling activity of each interaction of one of the 5 drug treatment subgroups against the activity in healthy donors (grey) and untreated

patients (orange). The colored thresholds show the upper quartile of each corresponding subgroup mean defining as signaling noise those interactions with signaling activity below that threshold.

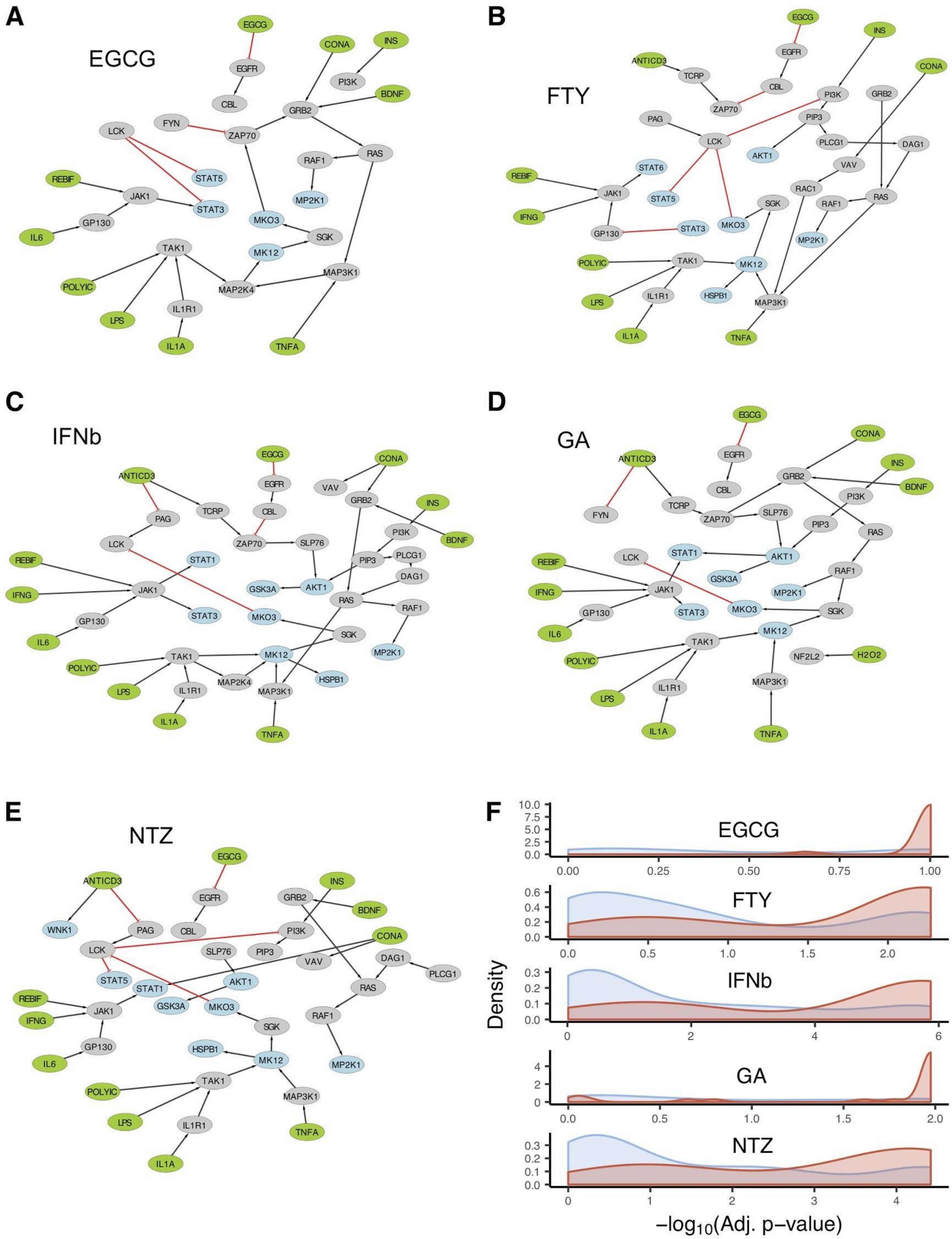
A
EGCG
EGCG
EGFR
CBL
CONA
INS
PI3K
BDNF
GRB2
FYN
ZAP70
LCK
RAF1
RAS
STAT5
REBIF
JAK1
STAT3
MP2K1
GP130
MKO3
SGK
IL6
TAK1
MK12
MAP3K1
POLYIC
MAP2K4
IL1R1
LPS
IL1A
TNFA
B
FTY
EGCG
INS
EGFR
ANTICD3
TCRP
CBL
CONA
PI3K
GRB2
ZAP70
PAG
PIP3
LCK
AKT1
PLCG1
DAG1
VAV
REBIF
STAT6
RAC1
JAK1
STAT5
RAF1
RAS
IFNG
SGK
MKO3
GP130
STAT3
MP2K1
POLYIC
TAK1
MK12
LPS
IL1R1
HSPB1
MAP3K1
IL1A
TNFA
C
IFNb
EGCG
CONA
ANTICD3
EGFR
VAV
GRB2
TCRP
CBL
INS
PAG
PI3K
BDNF
LCK
ZAP70
SLP76
PLCG1
REBIF
STAT1
PIP3
DAG1
IFNG
JAK1
GSK3A
AKT1
RAS
RAF1
STAT3
MKO3
GP130
SGK
IL6
MP2K1
TAK1
MK12
POLYIC
HSPB1
MAP2K4
LPS
IL1R1
MAP3K1
IL1A
TNFA
D
GA
EGCG
CONA
ANTICD3
EGFR
INS
GRB2
CBL
BDNF
FYN
TCRP
PI3K
ZAP70
SLP76
PIP3
RAS
LCK
STAT1
AKT1
REBIF
GSK3A
RAF1
IFNG
JAK1
MP2K1
GP130
STAT3
MKO3
SGK
IL6
TAK1
MK12
POLYIC
NF2L2
H2O2
IL1R1
MAP3K1
LPS
IL1A
TNFA
E
NTZ
EGCG
ANTICD3
INS
EGFR
GRB2
BDNF
WNK1
PAG
CBL
PI3K
CONA
LCK
SLP76
PIP3
VAV
DAG1
PLCG1
STAT5
AKT1
RAS
STAT1
REBIF
GSK3A
MKO3
RAF1
IFNG
JAK1
SGK
GP130
HSPB1
MK12
IL6
MP2K1
POLYIC
TAK1
MAP3K1
LPS
IL1R1
TNFA
IL1A
F
EGCG
FTY
IFNb
GA
NTZ
Density
−log10(Adj. p−value)
10.0
7.5
5.0
2.5
0.0
0.00
0.25
0.50
0.75
1.00
0.6
0.4
0.2
0.0
0.0
0.5
1.0
1.5
2.0
0.3
0.2
0.1
0.0
0
2
4
6
4
2
0
0.0
0.5
1.0
1.5
2.0
0.3
0.2
0.1
0.0
0
1
2
3
4

Figure S3. Signaling networks found for each MS-specific therapy. A-E) The models previously calculated for each patient were merged to reveal the common active pathways for the experimental drug EGCG and for each MS drug (FTY, IFNβ, GA and NTZ); **F)** The differentially phosphorylated proteins were overrepresented (x axis shows the –log10 adjusted p-value) in pathways predicted by modeling as a density score (red line: protein found in model for each corresponding therapy, blue line: absent in that model) and confirmed statistically (see main text).

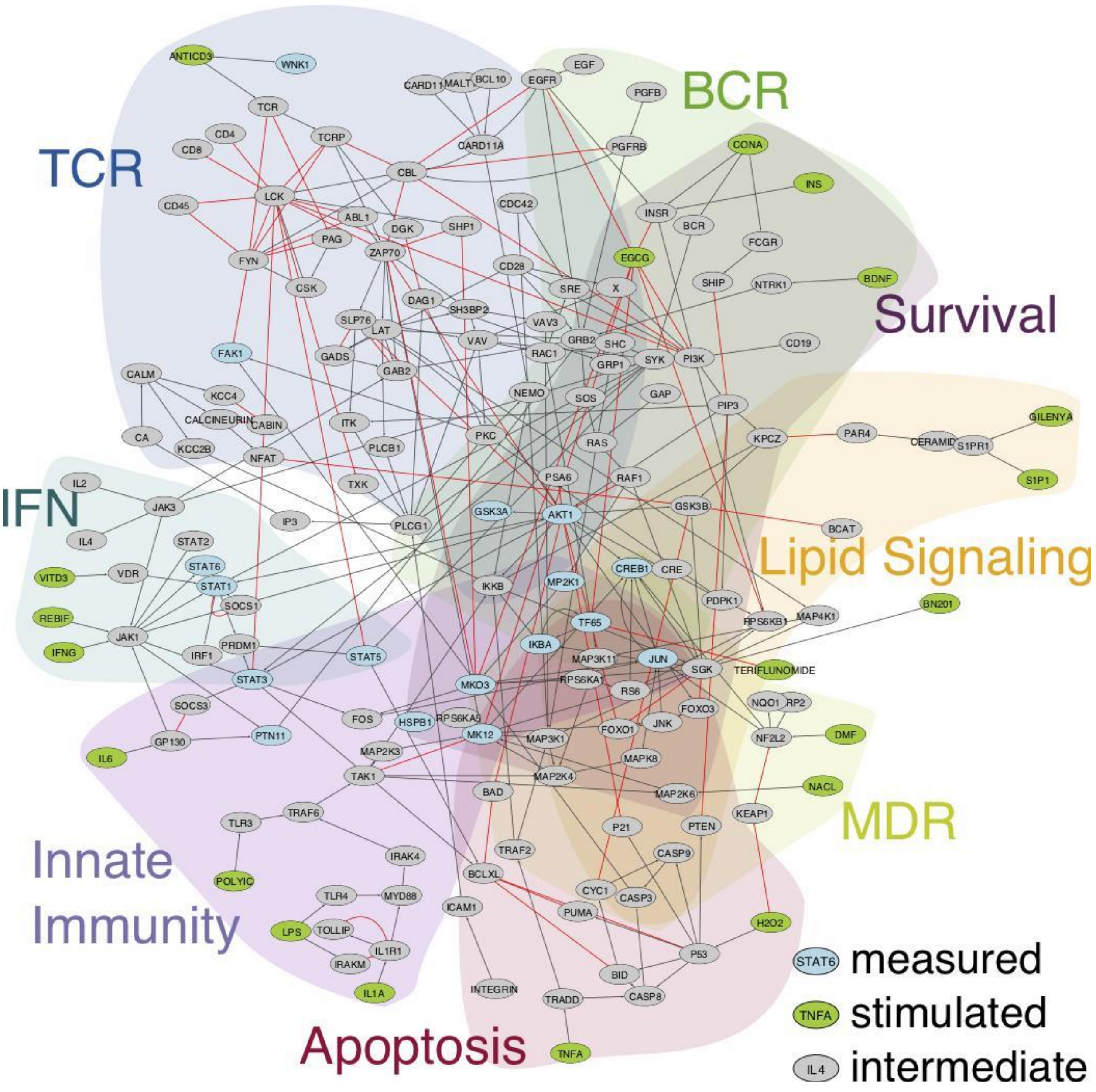

Figure S4. Reference-based curated MS- and immune-specific Prior Knowledge signaling Network (PKN). Shadows indicate the signaling pathways studied (interferon response (IFNβ), B-cell receptor (BCR) signaling, T- cell receptor (TCR) signaling, cellular survival and apoptosis, lipid signaling, innate immunity and multi-drug response (MDR) genes), including the crosstalk among them. Blue ovals: experimentally measured phosphoproteins; grey ovals: non-measured phosphoproteins and other molecules involved in signaling; green ovals: stimuli used in the *in vitro* assays; red lines: inhibitory reactions; black lines: activatory reactions.

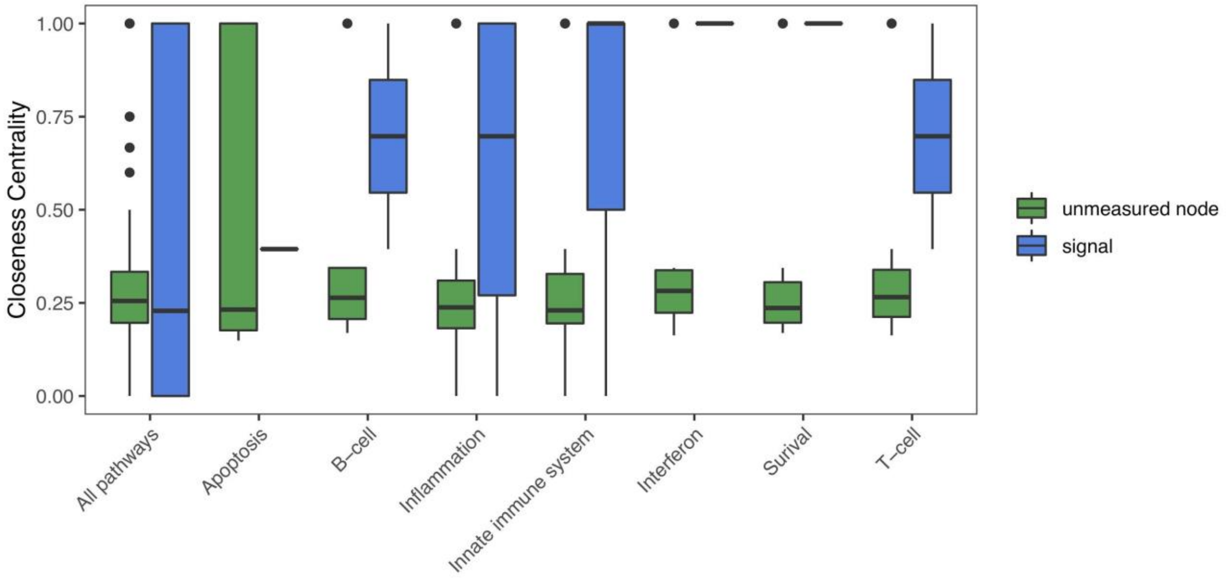

Figure S5. Signals experimentally measured are representative of Immune and MS pathways. Closeness centrality shows that efficiency for signal transduction across pathways (X axis) is higher in the network nodes (Figure S4) that were chosen as phosphoproteins to be measured than in most of those who were not selected. As negative control, closeness centrality is not higher in the median across pathways than across unmeasured nodes.

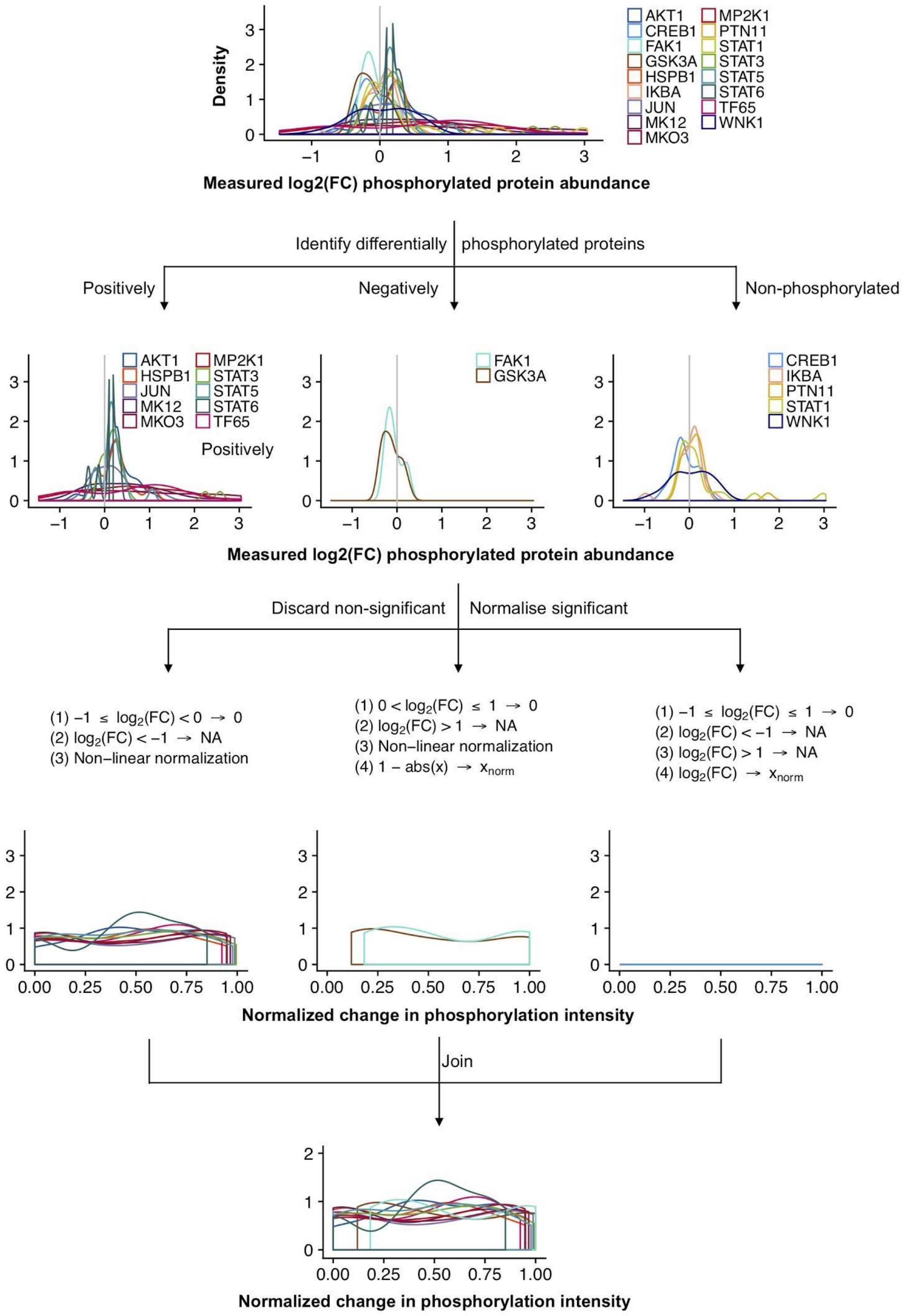
Density
Measured log2(FC) phosphorylated protein abundance
AKT1
CREB1
FAK1
GSK3A
HSPB1
IKBA
JUN
MK12
MKO3
MP2K1
PTN11
STAT1
STAT3
STAT5
STAT6
TF65
WNK1
Identify differentially phosphorylated proteins
Positively
Negatively
Non-phosphorylated
Positively
Measured log2(FC) phosphorylated protein abundance
Discard non-significant
Normalise significant
(1) −1 ≤ log2(FC) < 0 → 0
(2) log2(FC) < −1 → NA
(3) Non−linear normalization
(1) 0 < log2(FC) ≤ 1 → 0
(2) log2(FC) > 1 → NA
(3) Non−linear normalization
(4) 1 − abs(x) → xnorm
(1) −1 ≤ log2(FC) ≤ 1 → 0
(2) log2(FC) < −1 → NA
(3) log2(FC) > 1 → NA
(4) log2(FC) → xnorm
Normalized change in phosphorylation intensity
Join
Normalized change in phosphorylation intensity

Figure S6. Phosphoproteomics normalization pipeline. The graph shows the normalization algorithm developed to identify and select the significantly positive and negative phosphorylated measurements, and normalize them accordingly to allow Boolean modeling (see methods). For consistency, the data highlighted in Figure 2 (patient KI044) was used to visualize the normalization process.
